# Novel Electroactive Therapeutic Platforms for Cardiac Arrhythmia Management

**DOI:** 10.1002/advs.202500061

**Published:** 2025-02-14

**Authors:** Juwei Yang, Longfei Li, Yiran Hu, Zhou Li, Wei Hua

**Affiliations:** ^1^ The Cardiac Arrhythmia Center State Key Laboratory of Cardiovascular Disease National Clinical Research Center of Cardiovascular Diseases National Center for Cardiovascular Diseases Fuwai Hospital Chinese Academy of Medical Sciences and Peking Union Medical College Beijing 100037 China; ^2^ Beijing Institute of Nanoenergy and Nanosystems Chinese Academy of Sciences Beijing 101400 China

**Keywords:** cardiac arrhythmias, conductive systems, electroactive platforms, implantable devices, telemedicine

## Abstract

Electroactive platforms have gained significant attention for their ability to convert various types of energy into electrical signals, offering promising applications in diverse biomedical fields. In cardiovascular care, these platforms are increasingly valued for their innovative solutions in managing cardiac functions and disorders. By regulating electrical activity in the heart, electroactive platforms offer novel methods for managing abnormal heart rhythms. This review explores the latest advancements in electroactive systems, categorizing them based on their energy sources and mechanisms, such as self‐powered and conductive systems. It also highlights their applications in arrhythmia management, including monitoring, intervention, pacing, and repairing. Finally, the challenges, limitations, and future opportunities for clinical translation of these technologies are discussed.

## Graphical abstract

Electroactive platforms offer promising applications in cardiac arrhythmia. Based on their energy sources and mechanisms, electroactive platforms are categorized into i) direct electrical stimulation, ii) self‐powered electroactive systems, iii) physical stimuli‐mediated electroactive systems, and iv) conductive systems, and their applications in arrhythmia management, including monitoring, intervention, pacing, and repairing are highlighted.

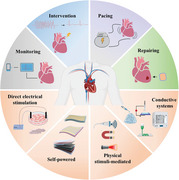

## 1 Introduction

Electrical signals serve as a crucial mode of communication in living organisms, maintaining the normal functions of various organs, including the heart^[^ ^1^ ^]^ and brain.^[^ ^2^ ^]^ As early as the 19th century, the phenomenon of bioelectricity attracted the attention of scientists. Subsequently, the development of techniques such as action potential recording^[^ ^3^ ^]^ and electrocardiography^[^ ^4^ ^]^ further revealed the key role of electrical signals in cardiac rhythm regulation. Bioelectricity is vital for maintaining cardiac electrical conduction, and when it fluctuates abnormally, it can lead to various arrhythmias,^[^ ^5^ ^]^ such as atrial fibrillation (AF) and ventricular arrhythmias, which may result in severe consequences like stroke, cardiac arrest, and sudden cardiac death (SCD). Epidemiological data indicate that the incidence of arrhythmias, including conduction abnormalities, significantly increases with age and body mass index.^[^ ^6^ ^]^ Globally, the mortality and complications associated with related diseases show an upward trend each year.^[^ ^7^ ^]^

To address the problems caused by arrhythmias, many approaches, including antiarrhythmic drugs (AADs), ablation procedures, and cardiac implantable electronic devices (CIEDs), have been developed and have made significant advancements in managing arrhythmias. For instance, by optimizing the dosage and combinations of AADs and developing novel AADs, safer and more effective therapies for atrial fibrillation have been created.^[^ ^8^ ^]^ Advances in ablation techniques, including novel energy sources and 3D mapping systems, have increased success rates and reduced recurrence rates.^[^ ^9^ ^]^ Furthermore, the introduction of new implantable devices, such as implantable cardioverter‐defibrillators (ICDs), implantable loop recorders (ILRs), and leadless pacemakers, has significantly improved patient survival rates and quality of life.^[^ ^10^ ^]^ However, with the increasing complexity of diseases and the growing demand for personalized care, the limitations of these methods have become more apparent, such as drug side effects,^[^ ^11^ ^]^ the possibility of a second recurrence of arrhythmia,^[^ ^12^ ^]^ and surgical complications,^[^ ^13^ ^]^ highlighting the necessity for alternative therapeutic approaches.

With advancements in materials science and biomedical engineering, electroactive materials have gradually been applied in the field of cardiovascular therapy. These materials endow high biocompatibility and flexible mechanical properties, allowing them to respond to/conduct electrical stimuli/convert other stimuli (such as mechanical stimulation, magnetic fields, light, etc.) into electrical signals, which can regulate cell activity and promote cell maturation, proliferation, and differentiation.^[^ ^14^ ^]^ By integrating electroactive materials with topological, chemical, or mechanical features, more multifunctional scaffolds or platforms can be constructed for sensing, therapeutic, and monitoring applications.^[^ ^15^ ^]^ Additionally, incorporating different bioactive components can be more beneficial in mimicking the biophysical properties of the extracellular matrix (ECM) can precisely regulate the cellular microenvironment,^[^ ^14^, ^16^ ^]^ enhancing myocardial repair and regeneration. These electroactive platforms have demonstrated their multifunctionality in various clinical settings and are widely used in neuromodulation,^[^ ^17^ ^]^ bone repair,^[^ ^18^ ^]^ and wound healing.^[^ ^19^ ^]^

**Figure 1:**
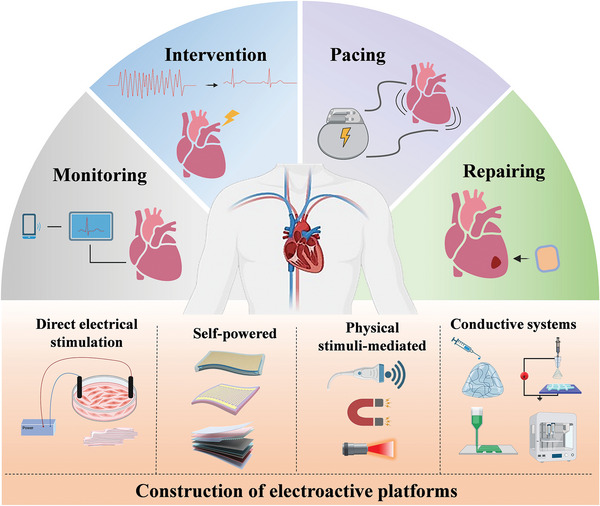
Schematic of electroactive platforms for cardiac arrhythmia applications and various construction methods.

Considering the importance and potential of platforms constructed based on electroactive materials for arrhythmia modulation, as well as the relatively limited number of reports associating electroactive materials and arrhythmias. This review aims to fill this gap by focusing on the application of electroactive platforms in the field of arrhythmias. We have summarized recent research advances regarding electroactive materials and their implementation in this area. We first provide an overview of the current status of arrhythmias, including the basic mechanisms of cardiac electrophysiology and existing treatment methods. Then the categories for the construction of electroactive platforms are presented, which include i) direct electrical stimulation, ii) self‐powered electroactive systems, iii) physical stimuli‐mediated electroactive systems, and iv) conductive systems. Afterward, the progress of these electroactive platforms in arrhythmias in recent years is summarized, focusing on monitoring, intervention, pacing, and repair of cardiac tissue (**Figure** **1**). Finally, the current limitations and challenges faced are discussed, and the future directions are also prospected, with the expectation of providing new insights into arrhythmia treatment protocols and advancing future therapeutic strategies.

## 2 Overview of Cardiac Electrophysiology

**Figure 2:**
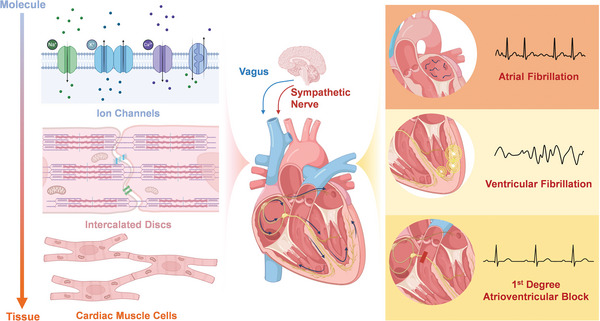
Illustration of the physiological and molecular basis of cardiac electrical activity, showing ion channels, intercalated discs, and neural regulation, along with examples of arrhythmias and their representative ECG signals.

A comprehensive understanding of cardiac electrophysiology is essential for elucidating the physiological mechanisms underlying cardiac electrical activity (**Figure** **2**). Examining these mechanisms provides critical insights into the underlying factors of arrhythmias and establishes a scientific basis for developing electroactive platforms and novel therapeutic strategies in this field.

### 2.1 Physiological Basis of Cardiac Electrical Activity

The primary function of cardiac electrical activity, governed by excitation‐contraction coupling (ECC), is to drive blood circulation. Action potentials are generated through the flow of ions, particularly Na⁺, K⁺, and Ca^2^⁺, across ion channels in the membranes of cardiac myocytes. In phase 0, rapid Na⁺ influx initiates depolarization, followed by an interplay between K⁺ and Ca^2^⁺ that shapes the plateau and repolarization phases. During phase 2, Ca^2^⁺ enters the cytoplasm, binding to ryanodine receptors on the sarcoplasmic reticulum to trigger calcium‐induced calcium release (CICR). This released Ca^2^⁺ binds to troponin, initiating cardiac muscle contraction. Cellular relaxation, in turn, requires efficient removal of Ca^2^⁺ from the cytoplasm to allow myocytes to return to their resting state. This precise balance of calcium influx and efflux is essential to maintain coordinated contraction and relaxation cycles in cardiac myocytes.^[^ ^20^ ^]^ Working cells maintain a stable membrane potential at rest through Na⁺−K⁺ ATPase and Na⁺−Ca^2^⁺ exchangers, whereas pacemaker cells (such as those in the sinoatrial node) exhibit spontaneous depolarization in phase 4 and generate rhythmic cardiac excitation.

Intercalated discs are complex structures that connect cardiac myocytes, composed of adhesion junctions, desmosomes, and gap junctions; these components play a crucial role in conducting electrical signals and coordinating mechanical forces, thereby facilitating synchronous contractions of the heart. Adhesion junctions are multi‐protein complexes that transversely connect adjacent myocyte myofibrils and actin, primarily transmitting mechanical forces and maintaining the integrity of the cellular structure.^[^ ^21^ ^]^ Desmosomes connect to other intracellular structures via intermediate filaments (or desmin), helping to maintain the appropriate positioning of cellular components during the vigorous contraction and relaxation of the heart.^[^ ^22^ ^]^ Gap junctions, formed by six connexin monomers, facilitate the rapid transfer of substances and electrical activity through the cytoplasm.^[^ ^23^ ^]^ Among them, connexin 43 (Cx43) is the most common subtype in working myocytes which is encoded by the GJA1 gene, while connexin 40 (Cx40) is predominantly expressed in the atria and conduction system.^[^ ^24^ ^]^

In addition to the coordination of individual cardiac myocytes and their intercellular structures, the heart relies on a complex conduction system to integrate and regulate overall electrical activity. This system includes the sinoatrial node (SAN), atrioventricular node (AVN), His bundle, right and left bundle branches, and Purkinje fibers. Normally, electrical impulses originate from spontaneous depolarization in the sinoatrial node and rapidly propagate through the atrial muscle, slowing down as they conduct through the interatrial bundle to the atrioventricular node, resulting in an atrioventricular delay (AV delay). This delay allows the atria and ventricles to contract in coordination, preventing excessively fast ventricular rates during rapid atrial arrhythmias, which could have severe consequences.^[^ ^25^ ^]^ The impulse continues down the His bundle, reaching the right and left bundle branches, further splitting into Purkinje fibers, and ultimately transmitting the electrical impulse to the ventricular myocytes through the intercalated discs.^[^ ^26^ ^]^ The conduction velocity in the bundle branches and Purkinje fibers is extremely fast, allowing for nearly simultaneous activation of all ventricular muscles. Through the functions of ion channels, maintenance of resting potentials, propagation of action potentials, and the support of intercalated disc structures, the heart operates as a functional syncytium, ensuring normal pumping function under different physiological states.

The autonomic nervous system (ANS) plays a crucial role in regulating cardiac electrical activity. The vagus nerve releases acetylcholine, acting on M2 muscarinic receptors on sinoatrial node cells, increasing potassium influx and decreasing cAMP levels, ultimately leading to decreased automaticity of the sinoatrial node and a slower heart rate.^[^ ^27^ ^]^ Additionally, activation of the vagus nerve can reduce conduction velocity in the atrioventricular node (negative dromotropic effect), preventing rapid atrial electrical activity from reaching the ventricles, thus preventing certain types of arrhythmias, such as rapid ventricular responses during atrial fibrillation.^[^ ^28^ ^]^ Conversely, sympathetic activation releases norepinephrine (NE), which binds to *β*1‐adrenergic receptors on cardiac cell membranes, increasing cAMP levels, enhancing the automaticity of sinoatrial node cells, and accelerating heart rate.^[^ ^29^ ^]^ Sympathetic stimulation also speeds up the conduction of electrical signals within the heart (positive dromotropic effect), particularly increasing conduction velocity at the atrioventricular node, reducing the risk of atrioventricular block, although it may promote the occurrence of arrhythmias in certain cases.^[^ ^30^ ^]^ Through these mechanisms, the autonomic nervous system ensures that the heart can flexibly adjust its electrical activity and pumping function according to the body's demands, maintaining normal operation. Dysfunction or abnormalities in any of these critical processes can precipitate arrhythmias,^[^ ^31^ ^]^ which in turn disrupt normal cardiac function and may give rise to a cascade of pathological consequences previously mentioned.

### 2.2 Current Curing Modalities

Technically arrhythmias can be categorized into two types: tachyarrhythmia and bradyarrhythmia. Although the mechanisms of these two types differ, both can lead to a decrease in the effective output of the heart, subsequently triggering a range of clinical symptoms. AF is the most prevalent type of tachyarrhythmia, closely related to abnormal electrical activity from multiple ectopic pacemaker sites within the atria. Bradyarrhythmia is often associated with dysfunction of the sinoatrial node or atrioventricular node, such as sinus bradycardia or atrioventricular block (AVB). Currently, there are various clinical approaches for treating arrhythmias, mainly including AADs, surgical interventions, and implantable device therapy.

AADs serve as the cornerstone of treatment for arrhythmias, particularly for patients with mild to moderate conditions. AADs work by modulating the electrical activity of the heart to restore normal rhythm. The traditional classification system is based on the Vaughan‐Williams scheme, which divides drugs into four main classes: Class I sodium channel blockers, Class II *β*‐blockers, Class III potassium channel blockers, and Class IV calcium channel blockers. Class I drugs (e.g., quinidine and lidocaine) inhibit fast sodium channels, thereby delaying depolarization in cardiac myocytes. Class II drugs (e.g., propranolol and metoprolol) reduce sympathetic nervous system activity, resulting in a slower heart rate. Class III drugs (e.g., amiodarone and sotalol) inhibit potassium channels, prolonging action potential duration (APD) and thereby suppressing early repolarization phenomena. Class IV drugs (e.g., verapamil and diltiazem) inhibit calcium channels, reducing conduction velocity in the atria and atrioventricular node. Although AADs are widely used in clinical practice, their potential for serious side effects and exacerbation of arrhythmias necessitates cautious use. The traditional classification system for AADs is based on the Vaughan‐Williams scheme, categorizing them into four main classes. Class I includes sodium channel blockers like quinidine and lidocaine; these drugs inhibit fast sodium channels, delaying depolarization in cardiac myocytes. Class II features *β*‐blockers such as propranolol and metoprolol, which reduce sympathetic nervous system activity and lead to a slower heart rate. In Class III, potassium channel blockers like amiodarone and sotalol prolong APD by inhibiting potassium channels, thereby suppressing early repolarization phenomena. Finally, Class IV consists of calcium channel blockers, including verapamil and diltiazem, that reduce conduction velocity in the atria and atrioventricular node through calcium channel inhibition. Despite their widespread use in clinical practice, AADs carry risks of serious side effects and potential exacerbation of arrhythmias, necessitating cautious administration.^[^ ^11^ ^]^

Catheter ablation, an important method for treating arrhythmias, has rapidly evolved since its introduction nearly 50 years ago.^[^ ^32^ ^]^ This technique utilizes catheters to deliver radiofrequency, cryoablation, laser, or the latest pulsed electric fields, targeting and destroying abnormal electrical conduction pathways or ectopic pacemaker sites to restore normal rhythm. Currently, the clinical efficacy of catheter ablation is widely recognized and applicable to various arrhythmias (e.g., AF, atrial flutter, supraventricular tachycardia), effectively reducing recurrence rates and lowering mortality associated with arrhythmias.^[^ ^33^ ^]^

CIEDs include permanent pacemakers (PPM), implantable cardioverter‐defibrillators (ICD), cardiac resynchronization therapy devices (CRT‐P/CRT‐D), and implantable loop recorders (ILR). Permanent pacemakers regulate the heart's contraction rhythm by providing stable electrical stimulation (ES), thereby restoring effective blood circulation in patients with sinus bradycardia or AVB. In recent years, physiological conduction system pacing techniques have led to breakthroughs that can more effectively restore the heart's electrical conduction mechanisms than traditional right ventricular pacing, thereby improving cardiac function and enhancing patient outcomes.^[^ ^34^ ^]^ In addition, ICDs provide anti‐tachycardia pacing (ATP) functions, suitable for patients at high risk for ventricular fibrillation (VF) or refractory ventricular tachycardia (VT). CRT devices correct inter‐ventricular or intra‐ventricular desynchrony, often used in heart failure patients with left bundle branch block. Furthermore, ILRs assist in the early diagnosis of arrhythmias by remote monitoring and recording abnormal heart rhythm events in real‐time.^[^ ^35^ ^]^

## 3 Construction of Electroactive Platforms

The development of electroactive platforms offers innovative solutions that can address certain limitations of existing treatment modalities while optimizing existing clinical treatment methods for arrhythmias. These platforms leverage electrical stimulation to provide precise cardiac activity modulation, thereby addressing traditional methods' shortcomings. This section will delve into the diverse methodologies involved in constructing these platforms.

### 3.1 Direct Electrical Stimulation

There are three primary methods for applying ES in cell culture.^[^ ^36^ ^]^ The first is direct coupling, where electrodes are directly attached to the tissue to deliver ES. This method offers the highest energy transfer efficiency and is widely utilized due to its simplicity, although it presents safety concerns and demands high electrode biocompatibility.^[^ ^37^ ^]^ Capacitive coupling involves placing two electrodes opposite each other, with the culture medium serving as a conductor, necessitating higher voltages to provide a uniform electric field.^[^ ^38^ ^]^ Last, inductive coupling utilizes conductive coils placed around the culture medium, generating a pulsed electromagnetic field to stimulate the cells.

As mentioned earlier, the heart's intrinsic electrical activity continuously stimulates cardiomyocytes at a frequency of 60–100 beats per minute, which is critical for their maturation and differentiation.^[^ ^39^ ^]^ In vitro ES can enhance the excitation‐contraction coupling mechanisms, thereby promoting cardiomyocyte maturation. Early studies demonstrated that ES aids in organizing neonatal rat cardiomyocytes on scaffolds and enhances cell‐cell connectivity.^[^ ^40^ ^]^ Another study found that ES of neonatal mouse cardiomyocytes significantly reduced APD, with increased expression of potassium channel‐related genes such as Kcnh2 and Kcnd2.^[^ ^41^ ^]^ Moreover, George et al. demonstrated that applying 2 Hz ES increased the expression of connexins (Cx43 and Cx40) in human stem cell‐derived cardiomyocytes. This enhancement improved the sarcomere structure and promoted cellular maturation, ultimately enhancing the cells' automaticity. Post‐stimulation, these cardiomyocytes continued to beat for up to two weeks and transmit electrical signals to neighboring cells.^[^ ^42^ ^]^ Furthermore, ES is not only essential for cardiomyocyte maturation but also promotes vascularization in engineered cardiac tissues.^[^ ^43^ ^]^ Among them, ES significantly enhanced endothelial cell elongation, migration, and interconnectivity, thus facilitating vascular network formation. This highlights the potential of ES as a regulatory factor in building vascularized cardiac tissues for clinical applications.

On the other hand, ES combined with mechanical stimulation can synergistically simulate the complex environment required for cardiomyocyte maturation. When neonatal rat cardiomyocytes were subjected to both mechanical stimuli and 4 Hz ES, it improved the force‐frequency relationship,^[^ ^44^ ^]^ a hallmark of mature ventricular cardiomyocytes critical for proper cardiac contraction. Moreover, this combination promoted the maturation of the T‐tubule system, increasing the number of T‐tubules and enhancing calcium handling and sarcoplasmic reticulum release capacity. While 2 Hz ES alone enhanced the contractility of engineered cardiomyocytes and upregulated several calcium‐handling proteins such as SERCA2 and RYR2. When combined with mechanical stress, cardiomyocytes displayed additional increases in contractility without altering tissue organization or cell size, indicating further maturation of the excitation‐contraction coupling mechanisms.^[^ ^45^ ^]^

Advancements in biomedical engineering have led to the emergence of novel ES platforms that integrate more sophisticated functionalities and offer broader applications. In one study, a biosensing platform integrating ES and automated video analysis was developed for large‐scale rhythmic regulation of cardiomyocytes.^[^ ^46^ ^]^ This system efficiently screened optimal ES conditions to modulate the state of ex vivo cardiomyocytes. Similarly, an ES system for the maturation of human induced pluripotent stem cell‐derived cardiac microfibers (CMFs) can stabilize CMF cultures by placing tissue between electrodes,^[^ ^47^ ^]^ thus ensuring uniform stimulation coverage across the entire CMF region. In addition, combining electrospun nanofibers with ES to design a microfluidic platform can provide multiple signal cues to immature cardiomyocytes, facilitating their transition to an adult phenotype.^[^ ^48^ ^]^ This device not only promoted anisotropy in cardiac tissue but also validated the role of ES in increasing the expression of tight junction proteins such as Cx‐43 and upregulating key genes like cardiac‐specific troponin I and *β*‐myosin heavy chain.

### 3.2 Self‐Powered Electroactive Systems

Traditional ES techniques have made significant strides in cardiac tissue engineering; however, they still face various limitations. External power sources often utilize rigid materials that are large and mechanically incompatible with biological tissues. For example, lithium‐iodine batteries, with Young's modulus of approximately 13 GPa, and lithium‐carbon monofluoride batteries, ≈100 GPa, significantly exceed the modulus of cardiac tissue (1–100 kPa).^[^ ^49^ ^]^ Such mismatches can result in inflammation and tissue damage.^[^ ^50^ ^]^ The complexity of ES parameters, such as current intensity, frequency, and duration, poses significant challenges for ensuring therapeutic efficacy and safety. Excessive current or suboptimal parameter settings can lead to overheating, tissue damage, and other adverse effects.^[^ ^51^ ^]^ Studies have shown that optimal electrical stimulation parameters for cardiac tissue engineering are ≈3 V cm^−1^ amplitude and 3 Hz frequency,^[^ ^52^ ^]^ while electrical energies exceeding 0.001 J may lead to adverse outcomes such as tissue overheating.^[^ ^53^ ^]^ These risks restrict the wider clinical application of ES technologies.

To address these issues, self‐powered electroactive systems have garnered increasing attention. These systems can autonomously generate power by harvesting energy from the biological environment of the human body, eliminating the need for external power sources and significantly enhancing the sustainability of bioelectronic platforms. Nanogenerators (NGs) are a crucial technology within self‐powered electroactive systems. Since their introduction in 2006,^[^ ^54^ ^]^ they have demonstrated extensive application potential. NGs can harness the biomechanical or bioelectric characteristics inherent to the human body, converting subtle energy from the biological environment into electrical power. Given that the heart continuously beats and generates significant mechanical and electrical energy, it offers a natural advantage for the application of NGs.^[^ ^55^ ^]^ Based on operational mechanisms, NGs can be categorized into the following types: piezoelectric nanogenerators (PENGs), triboelectric nanogenerators (TENGs), and pyroelectric nanogenerators, along with hybrid nanogenerators (H‐NGs). Due to limitations related to the working mechanisms, pyroelectric nanogenerators are not suitable for use as power sources in cardiovascular device implantation and are therefore not discussed in detail here.^[^ ^56^ ^]^ Representative types of NGs along with key structures and maximum outputs are summarized in **Table** **1**.

**Table 1:** Representative different types of NGs in the field of cardiac arrhythmias.

Types	Main materials	Key structures	Max. external output	Applications	Refs.
TENG	Spherical POM pellets PTFE	Micro‐nano structured surfaces; Freestanding mode	21.8 V	Self‐powered intracardiac pacemaker	[67]
TENG	Glass pellets with PTFE powder	Nanoparticles self‐adsorption	5.69 V	Cardiac contractility monitoring	[146]
TENG	Amine‐functionalized PVA‐NH2 and PFA	Synchronous stacked structure	136V	Self‐rechargeable cardiac pacemaker	[166]
TENG	Leaf‐vein patterned PVDF	Unique double‐spacer	21.98 mV	Sensing and repairing infarcted myocardium	[186]
PENG	PVDF	Wearable wristband design	0.75 V	AF monitoring	[60]
PENG	Porous P(VDF‐TrFE)	Helical structure self‐assembly	2.1 V	Cardiac energy harvesting and sensing	[61]
PENG	P(VDF‐TrFE)/BaTiO3	Layer‐by‐layer assembly	84 V	Self‐powered vagal neuromodulation	[165]
HENG	PTFE/PVDF	Multilayer structure	14.8 V		[68]

**Figure 3:**
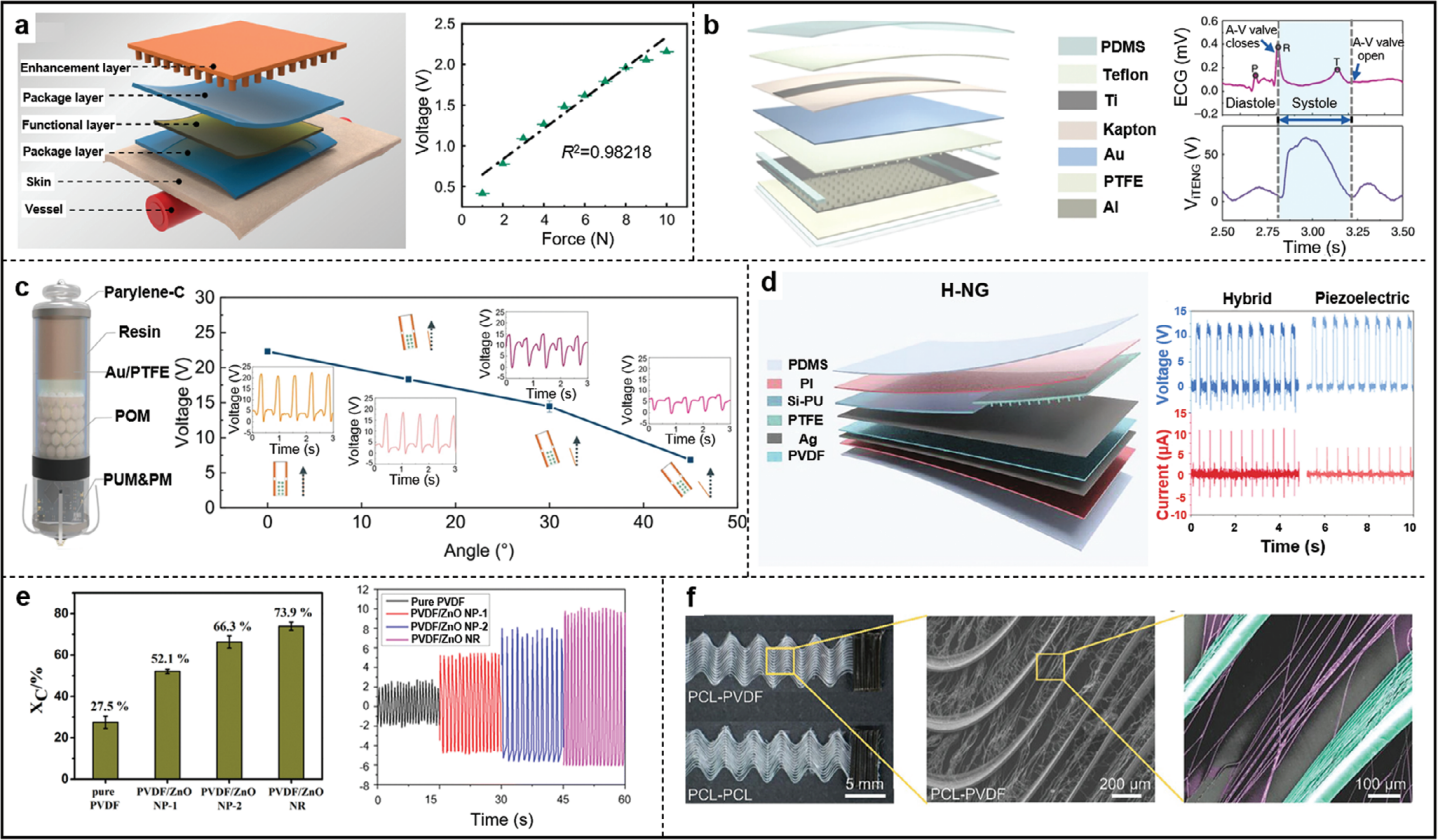
a) Schematic of the sensor structure and its linear force‐to‐voltage response. Reproduced with permission.^[^ ^60^ ^]^ Copyright 2023, Springer Nature. b) Schematic structure of the iTENG and the output open‐circuit voltage in vivo and simultaneously recorded ECG signals showing consistency. Reproduced with permission.^[^ ^66^ ^]^ Copyright 2019, Springer Nature. c) Schematic illustration of the energy harvesting unit (EHU) and Voc of EHU when working under different tilt angles. Reproduced with permission.^[^ ^67^ ^]^ Copyright 2024, Springer Nature d) Structure of H‐NG and improved performance of Hybrid‐NG than the piezoelectric module. Reproduced with permission.^[^ ^68^ ^]^ Copyright 2022, Elsevier. e) Crystallinity and piezoelectric output of different PVDF/ZnO nanofiber membranes. Reproduced with permission.^[^ ^73^ ^]^ Copyright 2024, The Royal Society of Chemistry. f) Representative images of PCL‐PVDF and PCL‐PCL scaffolds. Reproduced with permission.^[^ ^78^ ^]^ Copyright 2024, Wiley‐VCH.

PENGs convert mechanical energy into electrical energy based on the inherent properties of piezoelectric materials. When an external force is applied to these materials, a polarization phenomenon occurs, leading to the separation of positive and negative charge centers and generating a potential difference that drives free electrons through an external circuit, enabling energy conversion. Common piezoelectric materials include inorganic compounds such as zinc oxide (ZnO), barium titanate (BaTiO₃), and lead zirconate titanate (PZT), as well as organic piezoelectric materials like polyvinylidene fluoride (PVDF) and its derivatives, such as P(VDF‐TrFE).^[^ ^57^ ^]^ Moreover, biodegradable piezoelectric biomaterials like amino acids, peptides, and polylactic acid are increasingly utilized in the medical field.^[^ ^58^ ^]^ In 2010, Li et al. demonstrated the feasibility of harvesting biomechanical energy in vivo by implanting a single ZnO nanowire into the diaphragm of rats to capture energy from respiratory and cardiac movements, validating the potential of PENGs for collecting biomechanical energy in living organisms for the first time.^[^ ^59^ ^]^ More recently, the design and performance of PENG devices have improved significantly. A wearable piezoelectric wristband based on PVDF piezoelectric films combined with 3D‐printed reinforcement layers to optimize the collection of pulse wave signals, significantly increasing energy conversion efficiency (**Figure** **3**a).^[^ ^60^ ^]^ The addition of a hydrogel layer not only improved skin adhesion but also enhanced signal stability, ultimately yielding a pulse wave signal of up to 1.4 V with a favorable signal‐to‐noise ratio (SNR = 37 dB). Similarly, pacing wires based on multi‐layered porous PVDF‐TrFE piezoelectric films can harvest mechanical energy from heart contractions through bending and twisting motions.^[^ ^61^ ^]^ This design optimizes the deformation response of the films, effectively converting mechanical energy into electrical power and extending the battery life of pacemakers by ≈20%.

TENGs generate power through the electrostatic induction effect that occurs when two different materials come into contact and subsequently move relative to each other. This electrostatic phenomenon induces a redistribution of charges within the conductors, establishing a potential difference that propels electron flow in the external circuit, enabling efficient energy conversion. TENGs typically employ a combination of polymers and metals or different polymers to construct triboelectric layers and electrodes, including polymers such as polytetrafluoroethylene (PTFE), polydimethylsiloxane (PDMS), and polyimide (including Kapton), along with metallic electrodes like aluminum foil.^[^ ^62^ ^]^ TENGs have demonstrated significant potential in personalized health monitoring and portable therapeutic devices, owing to their low cost and flexible structures, thus becoming one of the most widely used methods for harvesting mechanical energy.^[^ ^63^ ^]^ For example, TENG can harvest biomechanical energy generated during respiration and further power a prototype pacemaker.^[^ ^64^ ^]^ Additionally, Ma et al. introduced an integrated, self‐powered, multifunctional implantable triboelectric active sensor (iTEAS).^[^ ^65^ ^]^ Under varying heart rates, the peak voltage of the sensor achieved a high level of synchronization with the R‐wave in the electrocardiogram. Ouyang et al. reported a symbiotic cardiac pacemaker positioned between the pericardium and the heart, utilizing a corona discharge method to increase the charge density on the PTFE surface (Figure 3b).^[^ ^66^ ^]^ A 3D elastic sponge (EVA) serves as a spacer, while a memory alloy keel functions as a supporting structure, both of which increase the effective contact area and further enhance the output performance of the TENG. As shown in Figure 3c, a self‐powered cardiac pacemaker (SICP) was designed featuring a capsule structure anchored at the apex of the heart.^[^ ^67^ ^]^ The capsule's interior is filled with polyoxymethylene (POM) particles that reciprocate on the PTFE membrane during heart motion, generating electrical energy. Furthermore, the team optimized the micro‐nano structure of the POM and PTFE surfaces through inductively coupled plasma (ICP) etching, significantly increasing the contact area and enhancing energy conversion efficiency, achieving an open‐circuit voltage of up to 6.0 V.

H‐NGs consist of at least two different NG types, allowing for the simultaneous utilization of multiple energy forms to improve performance. Sun et al. designed a closed‐loop vagus nerve stimulation system (LL‐VNS) based on H‐NGs, demonstrating significant innovations in device structure and performance (Figure 3d).^[^ ^68^ ^]^ This device is placed on the skin and features a unique “switch” design that semi‐separates the triboelectric and piezoelectric layers, serving both as a spacer for the triboelectric module and effectively reducing the risk of unintended stimulation due to accidental contact. With the incorporation of the triboelectric module, the output voltage increased by 110% and the current by 210%, greatly enhancing electron transfer efficiency. Additionally, H‐NGs exhibited excellent mechanical response linearity, maintaining stable performance under pressures ranging from 5 to 20 N and showing negligible performance degradation during a 10 000 s operational test, indicating their outstanding durability. Inspired by the structure of leaf veins, a flexible and transparent H‐NG that combines triboelectric, piezoelectric, and thermoelectric functionalities was developed by arranging silver nanowires into a leaf vein‐like network.^[^ ^69^ ^]^ This H‐NG can be affixed to various parts of the human body, simultaneously harvesting energy from mechanical and thermal sources for real‐time monitoring of various physiological signals.

Like piezoelectric nanogenerators, piezoelectric materials can generate electrical signals in response to localized mechanical deformation, an advantageous property for cardiac tissue. Moreover, implantable piezoelectric scaffolds can harvest the biomechanical forces generated by the spontaneous contraction of cardiac tissues for effective electrical stimulation therapy.^[^ ^70^ ^]^ Inorganic piezoelectric materials, including BaTiO_3_, ZnO, and some piezoelectric ceramics are used primarily as piezoelectric additives.^[^ ^71^ ^]^ Piezoelectric polymers are mainly PVDF and its copolymer P(VDF‐TrFE), polyhydroxybutyrate‐co‐hydroxyvalerate (PHBV), poly(*L*‐lactic acid) (PLLA), collagen, and silk protein.^[^ ^72^ ^]^ Scaffolds fabricated with these piezoelectric materials can spontaneously generate biomimetic bioelectricity to perform cardiac ECM‐like functions.

PVDF and its derivatives are widely used in cardiac patches due to their high piezoelectricity, which is mainly derived from their *β*‐phase. Conventional doping such as nanostructured ZnO can act as a nucleating agent to induce a crystal phase transition of PVDF from the *α*‐phase to the *β*‐phase, thus providing precise sensitivity to record blood pressure fluctuations in the distal and proximal parts of the cardiovascular system in real‐time.^[^ ^73^ ^]^ The incorporation of ZnO nanoparticles (ZnO NP) and multiwall carbon nanotubes can also lead to a significant increase in the output voltage of P(VDF‐TrFE) films.^[^ ^74^ ^]^ The doping of MXene and boron nitride nanosheets has similar positive effects.^[^ ^75^ ^]^ Electrospinning technology can also facilitate the formation of the *β*‐phase through strong electric fields and in‐situ polarization. Compared to the random fiber scaffold, the aligned P(VDF‐TrFE) fiber scaffold has a more significant *β*‐phase and exhibits a stable piezoelectric output that matches the mechanical load. Undifferentiated human induced pluripotent stem cells (hiPSCs) were cultured on the scaffolds for 40 days, and the troponin T and Cx43 showed high expression.^[^ ^76^ ^]^ As shown in Figure 3e, compared to ZnO NP, ZnO NR doped with PVDF (PVDF/ZnO NR) has excellent piezoelectric output voltage and current density. The obtained PVDF/ZnO NR was utilized as a piezoelectric working element implanted into the cardiovascular wall of the porcine heart and femoral arteries, where it can accurately monitor and record the micro‐pressure changes of various physiological states.^[^ ^73^ ^]^ Similar studies have demonstrated that piezoelectric scaffolds can promote cardiomyocyte maturation by increasing the expression of total Cx43 or phosphorylated Cx43, Ca^2+^ treatment properties, and ion channel expression.^[^ ^77^ ^]^ Figure 3f demonstrates the synergistic effect of mechanical/piezoelectric stimulation, where PVDF microfibers are used for piezoelectric stimulation and magnetic PCL/Fe_3_O_4_ microfibers are used as mechanical support, while deformation of the snake‐shaped structured scaffold can be controlled by external magnets, thus contributing to the piezoelectric microenvironment provided for CMs growth.^[^ ^78^ ^]^ Under dynamic mechanical stimulation, CMs on piezoelectric PCL‐PVDF scaffold exhibited hyper‐maturation with improved calcium transients and up‐regulated associated maturation genes compared to non‐piezoelectric PCL‐PCL scaffold. Until now, less work has been reported on other piezoelectric polymer scaffolds, which may be related to their low electromechanical coupling coefficients. However, it is foreseen that their good biocompatibility, processability, and excellent flexibility will further broaden the field of diagnosis and repair of cardiac tissue.

### 3.3 Physical Stimuli‐Mediated Electroactive Systems

In dealing with the power supply problem of conventional implantable cardiac patches, in addition to the above‐mentioned NGs, which have been developed by utilizing the body's mechanical energy,^[^ ^79^ ^]^ different energy harvesting techniques have been developed to modulate the physiological function of cardiac tissue by utilizing the material's inherent ability to respond to different exogenous stimuli (e.g., ultrasound, magnetic field, and light) to generate electrical stimuli.

**Figure 4:**
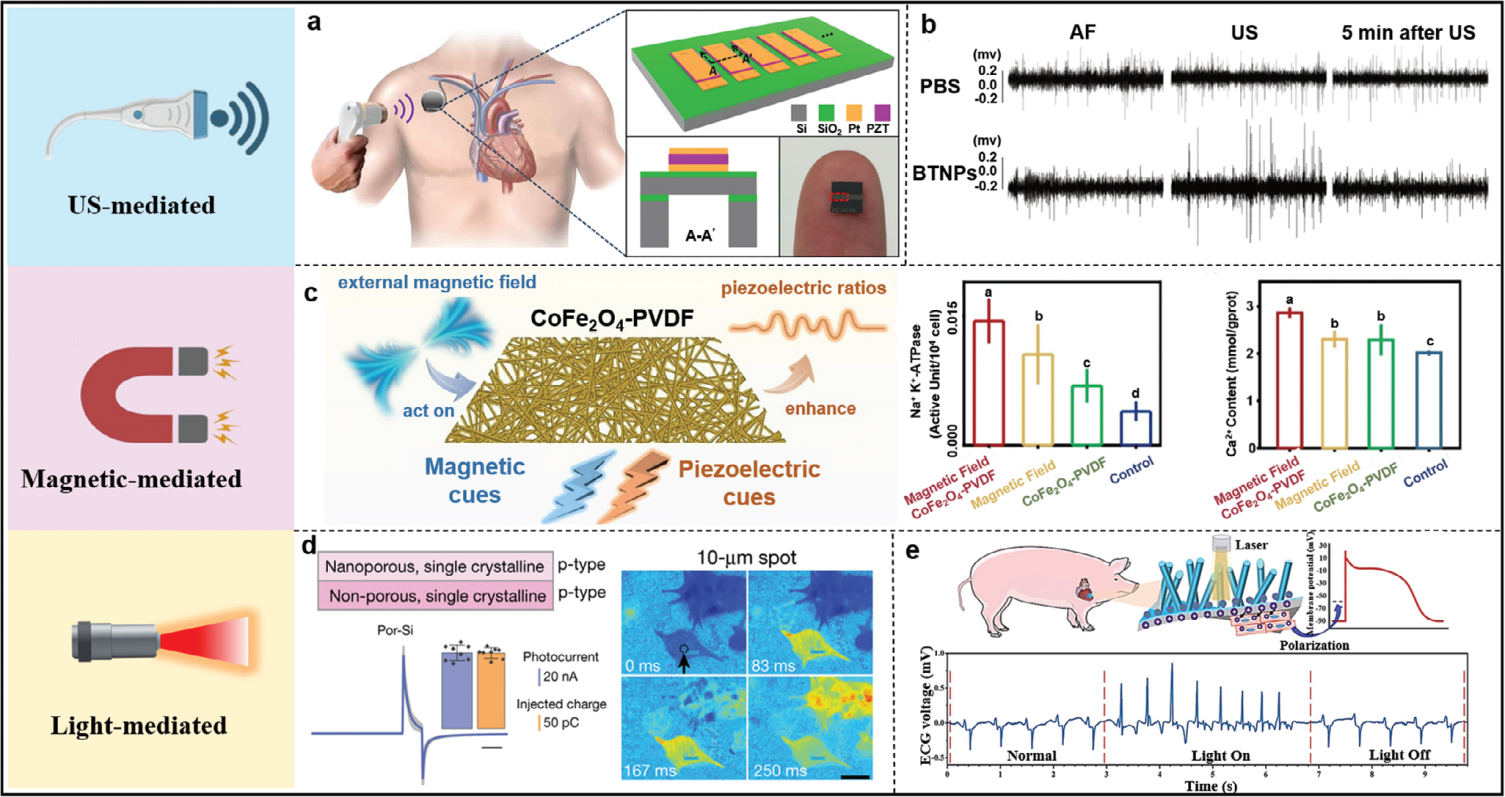
a) Schematic of a piezoelectric ultrasonic energy harvester integrated with a pacemaker and powered by ultrasound. Reproduced with permission.^[^ ^81^ ^]^ Copyright 2016, Open Access. b) Neural activation recordings from the IRGP in PBS and BTNPs group. Reproduced with permission.^[^ ^82^ ^]^ Copyright 2023, The Royal Society of Chemistry. c) Schematic representation of myocardial repair based on magnetoelectric synergistic and PVDF molecular chain conformation, as well as sodium‐potassium pump activity and intracellular calcium level measurements. Reproduced with permission.^[^ ^85^ ^]^ Copyright 2024, Elsevier. d) Schematic of Por‐Si device design with photocurrent magnitude and calcium influx and propagation with 10 µm spot stimulation. (Scale bar = 50 µm). Reproduced with permission.^[^ ^89^ ^]^ Copyright 2024, Springer Nature. e) Schematic diagram of cardiac pacing and ECG voltage signals recorded before, during, and after light stimulation. Reproduced with permission.^[^ ^90^ ^]^ Copyright 2020, Wiley‐VCH.

Ultrasound can deeply penetrate and focus on tissues, inducing biophysical effects such as cavitation and thermal effects.^[^ ^80^ ^]^ Utilizing acoustic energy transfer to convert ultrasound‐generated energy into electrical energy through a piezoelectric collector can be used to self‐power implantable devices. For example, a piezoelectric ultrasonic energy harvester prepared based on an array of lead zirconate titanate diaphragms can power various implantable biomedical devices by adjusting the ultrasound intensity (**Figure** **4**a), which can be used to power multiple implanted biomedical devices.^[^ ^81^ ^]^ BaTiO_3_, a classical piezoelectric material, can also generate a highly efficient electromagnetic field. BaTiO_3_ nanoparticles (BTNPs) encapsulated in ethylene glycol‐chitosan could generate electric fields under ultrasound treatment, and BTNPs reduced ventricular rate in sinus rhythm and AF models after ultrasound stimulation of the cardiac inferior right ganglionic plexus (IRGP), suggesting that ultrasound treatment of BTNPs activates the inward flow of Ca^2+^ from the extracellular space to stimulate the IRGP neurons and allows for the control of ventricular rate remotely and safely to control ventricular rate (Figure 4b).^[^ ^82^ ^]^

Pre‐exposure of the heart to a weak magnetic field reduces infarct size as revealed in previous studies.^[^ ^83^ ^]^ Composite multiferroic materials have been more widely studied than purely ferromagnetic materials, which usually consist of piezoelectric and magnetostrictive materials, and are essentially the synergistic result of magnetostrictive effects (magnetic/mechanical) and piezoelectric effects (mechanical/electrical).^[^ ^84^ ^]^ In other words, in a magnetic field, the magnetostrictive material first undergoes a shape change and endows the neighboring piezoelectric material with strain, generating/enhancing an electrical stimulation. Cobalt ferrite CoFe_2_O_4_ is one of the widely studied classes. After doping with CoFe_2_O_4_, the *β*‐phase, and crystallinity of PVDF were substantially improved, which effectively enhanced the piezoelectric effect of PVDF. Importantly, the magnetoelectric signals generated by the CoFe_2_O_4_/PVDF nanofiber scaffold can enhance sodium‐potassium (Na‐K) enzymes and increase intracellular Ca^2+^ (Figure 4c), which triggers cardiomyocyte contraction and overcomes the non‐conductance of damaged cardiomyocytes.^[^ ^85^ ^]^ The remote manipulation and non‐invasive nature of magnetic stimulation have also led to positive effects on neuromodulation, and with the potential to treat cardiovascular disease through autonomic neuromodulation, vagus nerve stimulation (VNS) has been demonstrated to be beneficial for cardiac repair after MI by enhancing parasympathetic drive.^[^ ^86^ ^]^ Hydrogels loaded with superparamagnetic iron oxide (SPIO) nanoparticles enable precise magnetic stimulation of individual vagus nerves, thus demonstrating the potential to improve MI through magnetic modulation of the nervous system.

Optoelectronic signals and related biological interfaces have been a hot topic of research. As familiar as optogenetics (which combines optics and genetics to achieve spatially and temporally precise activation or inhibition of target neurons) is similar but different,^[^ ^87^ ^]^ photoelectric conversion emphasizes the generation of electrical currents in response to stimulation by an external light source. Few materials endow this property, including silicon, graphene, and 𝝅‐conjugated polymers.^[^ ^88^ ^]^ As shown in Figure 4d, the analysis of four silicon‐based monolithic photovoltaic devices reveals that the silicon structure with porous heterojunction (Por‐Si) has high spatial resolution photocurrent tunability. This can be attributed to the fact that electrons diffuse as minority carriers in the p‐type nanoporous Si layer, the porous structure shortens the solid‐state diffusion length, thus providing optimal localization of the cathode photostimulation.^[^ ^89^ ^]^ By adjusting the light spot to 10 µm, the area of excited cells was successfully reduced to a single cell within a specific exposure time (Figure 4d). This study strongly confirms that within the range of photostimulation, cells initiate intercellular Ca^2+^ propagation and spreading. Hydrogenated amorphous silicon and radial p‐i‐n junctions grown on silicon nanowires enable the construction of a flexible self‐powered optoelectronic cardiac stimulator in which radial p‐i‐n junctions are exposed on the top surface.^[^ ^90^ ^]^ Under 650 nm laser irradiation, the optoelectronic device adheres well to the surface of the porcine heart and produces a peak ECG that changes significantly from the natural state (heart rate increases from 101 to ≈128 beats per minute) and returns to the natural heart rate when the light is removed (Figure 4e). PEDOT: PSS and polyalkylthiophene (P3HT) have advantages over crystalline Si for flexible applications.^[^ ^91^ ^]^ For example, a photoelectronic interface integrating PEDOT: PSS and hydrogel can simultaneously enable efficient, flexible, and safe cellular photostimulation. It can stimulate individual hippocampal neurons and control the beating frequency of cardiomyocytes,^[^ ^88^ ^]^ providing an opportunity to realize non‐invasive and reliable photoelectronic implants.

These physical stimuli (ultrasound, magnetic field, and light) provide adaptable and stable power output by delivering exogenous energy outside the body with various unique scaffolds. This non‐invasive therapy offers an alternative solution for maintaining sustained power to cardiac tissue.

### 3.4 Conductive Systems

Based on the concept of synchronized contraction of cardiac tissues triggered by electrical impulses,^[^ ^92^ ^]^ the matching in modulus, and the conformability of cardiac tissues, the electroactive platform mediated by conductive materials has been widely investigated.^[^ ^93^ ^]^ Conductive scaffolds not only provide physical support but also can be able to mimic the electrical conduction properties of the natural myocardium.^[^ ^70^ ^]^ They facilitate the rapid propagation of electrical signals within cardiomyocytes and interact with myocardial tissues through electrical integration, thus promoting functional maturation and tissue repair of cardiomyocytes.^[^ ^94^ ^]^ The review of the types of conductive materials (carbon‐based nanomaterials, metal‐based biomaterials, and conductive polymers) has been well described,^[^ ^95^ ^]^ so this section will display the application of various conductive systems on cardiac tissue according to the types of scaffolds constructed by different techniques, which can be categorized into hydrogel scaffolds, nanofiber scaffolds, 3D printing scaffolds, and composite‐scaffolds.

**Figure 5:**
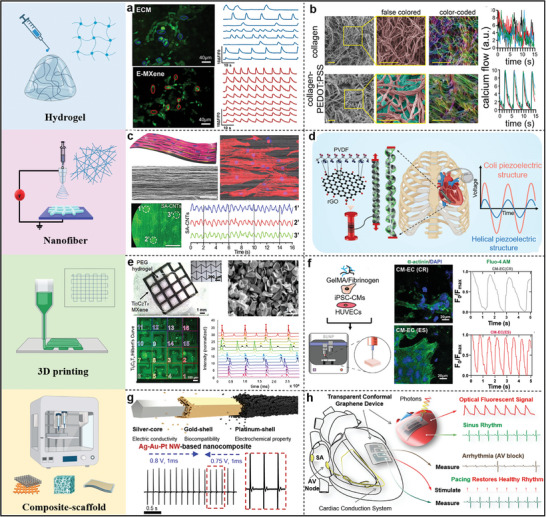
a) Calcium transients and corresponding frequency signatures of hiPSC‐CMs cultured in ECM and E‐MXene hydrogels. Reproduced with permission.^[^ ^102^ ^]^ Copyright 2024, Elsevier. b) False‐color region‐of‐interest images of collagen and collagen‐PEDOT:PSS hydrogels and corresponding intracellular calcium changes. Reproduced with permission.^[^ ^107^ ^]^ Copyright 2024, Wiley‐VCH. c) Schematic diagram of the simulated myocardial structure of SA‐CNTs and its representative SEM images, and CMs aggregates on SA‐CNTs showed regular, stable, and synchronized contraction after 4 d of incubation. Reproduced with permission.^[^ ^119^ ^]^ Copyright 2017, Wiley‐VCH. d) Schematic of PVDF/rGO nanocomposite scaffold fabricated by melt spinning for biomedical applications.^[^ ^121b^ ^]^ Copyright 2024, The Royal Society of Chemistry. e) An overview of Ti_3_C_2_T_x_‐PEG composite hydrogel and calcium flux delay recordings. Reproduced with permission.^[^ ^122b^ ^]^ Copyright 2022, Elsevier. f) Immunostaining of CM‐EC (CR) and CM‐EC (ES) cardiac constructs for the muscle proteins 𝛼‐actinin (green) and DAPI (blue) on day 24, and analysis of representative calcium transient profiles. Reproduced with permission.^[^ ^123^ ^]^ Copyright 2023, Wiley‐VCH. g) Schematic diagram of Ag‐Au‐Pt NW and surface electrocardiogram of rats after pacing with Ag‐Au‐Pt NW/Pt NP nanocomposite electrodes. Reproduced with permission.^[^ ^127^ ^]^ Copyright 2023, American Chemical Society. h) Schematic illustration of the multifunction of graphene biointerfaces. Reproduced with permission.^[^ ^134^ ^]^ Copyright 2023, Wiley‐VCH.

Compared to conventional metal conductive materials, hydrogels are better matched to the modulus of cardiac tissue (Young's modulus of ≈100 kPa),^[^ ^96^ ^]^ thereby effectively reducing the risk of local inflammation and scar formation. The most widely known one is the hydrogel incorporating conductive agents, including metal conductive substances and carbon‐based conductive substances such as gold nanorods/nanoparticles,^[^ ^97^ ^]^ carbon nanotubes (CNT),^[^ ^98^ ^]^ graphene oxide (GO),^[^ ^99^ ^]^ MXene,^[^ ^100^ ^]^ and others.^[^ ^101^ ^]^ Gelatin methacrylate (GelMA) hydrogels referenced with conductive gold nanorods (GNRs) can combine electrical stimulation and topographic cues to mimic myocardial function and exhibit consistent heart rate change responses to external stimuli.^[^ ^97b^ ^]^ Antioxidant/conducting hydrogels (E‐MXene) were prepared by embedding MXene nanosheets (Ti_3_C_2_) into extracellular matrix (ECM) hydrogels. As shown in **Figure** **5**a, hiPSC‐CMs on E‐MXene hydrogels showed higher frequency synchronized calcium signals than on ECM hydrogels. It was suggested that the incorporation of conductive MXene facilitated the electrical signal transmission between hiPSC‐CMs and effectively assisted the synchronized contraction of hiPSC‐CMs.^[^ ^102^ ^]^ Meanwhile, some polymers, mainly polyaniline (PANI),^[^ ^103^ ^]^ polypyrrole (PPy),^[^ ^104^ ^]^ and poly(3,4‐ethylenedioxythiophene) (PEDOT)^[^ ^105^ ^]^ can be used as hydrogel substrates as conductive patches to restore myocardial function. The presence of PEDOT:PSS in hydrogels has been demonstrated to be effective in improving the maturation and beating characteristics of cardiomyocytes, resulting in enhanced beat frequency, faster contraction rates, and prevention of arrhythmias.^[^ ^106^ ^]^ PEDOT:PSS improves the micromorphology and electrical conductivity of collagen hydrogels while improving cardiomyocyte electrical coupling, and the calcium waves of collagen‐PEDOT:PSS hydrogels are oriented and homogeneous.^[^ ^107^ ^]^ Moreover, compared with the injection of collagen hydrogel, which had no significant effect on the incidence of ventricular tachycardia (VT) after myocardial infarction, collagen‐PEDOT:PSS hydrogel significantly reduced the incidence of VT to 25% (Figure 5b). To address the invasiveness of cardiac patch implantation and deformation compliance, the diamond‐shaped patch with minimally invasive implantable injectability and shape memory properties was developed to eliminate atrial fibrillation.^[^ ^108^ ^]^ Poly‐3‐amino‐4‐methoxybenzoic acid gelatin (PAMB‐G) is another conductive polymer that has been shown to reduce pacemaker stimulation myocardial threshold voltage,^[^ ^109^ ^]^ shorten AF duration, and improve post‐AF recovery.^[^ ^110^ ^]^ Injection of PAMB‐G hydrogel into the scar area of rats after myocardial infarction improves the propagation of electrical impulses and synchronizes cardiac contraction, thereby preserving ventricular function and reducing arrhythmias.^[^ ^111^ ^]^ Further, loading active substances such as drugs/factors on the conductive hydrogel can modulate the microenvironment and remodel myocardial function.^[^ ^112^ ^]^ For instance, α‐tocopherol reduces oxidative stress‐induced apoptosis, prevents macrophage activation and pro‐inflammatory cytokine release, and facilitates the reversal of local myocardial oxidative damage.^[^ ^112a^ ^]^ Besides that, ion‐conductive hydrogels are also interesting because of their mechanical stability, high mechanical deformation sensing ability, and biocompatibility.^[^ ^113^ ^]^ For example, self‐repairing ionic hydrogel, prepared by introducing polyacrylic acid (PAA) into the hydrogel matrix, showed more pronounced directional sarcomeres than hydrogels with common conductive substrates. It also demonstrated attenuation of left ventricular remodeling and restoration of cardiac function in vivo.^[^ ^114^ ^]^

Electrospun fiber scaffolds have the advantages of porousness, adjustable mechanical properties, good biocompatibility, and customizable characteristics,^[^ ^115^ ^]^ which can be further combined with electrical signals to remodel the cardiac electrical microenvironment. Based on this, some conductive substances (CNT, rGO, nanosilver, etc.)^[^ ^116^ ^]^ were added to the electrospinning system or themselves (PPy, PANI, PEDOT:PSS, etc.)^[^ ^117^ ^]^ were prepared as nanofibers. For example, when electrical stimulation was applied to the CNT‐doped nanofiber scaffold promoted the differentiation of mesenchymal stem cells (MSCs) into cardiomyocytes and significantly increased the expression of cardiac markers, such as Nkx2.5 and troponin I.^[^ ^118^ ^]^ Furthermore, the addition of different concentrations of CNT significantly regulated the viability, alignment, and contractile activity of cardiomyocytes on the nanofiber scaffolds.^[^ ^116a^ ^]^ To mimic the hierarchical aligned structure in natural myocardium, super‐aligned carbon nanotube sheets (SA‐CNTs) were prepared for cultured cardiomyocyte arrangement morphology.^[^ ^119^ ^]^ Cells on SA‐CNTs exhibited regular and synchronized contractions compared to irregular, asynchronous contracting controls (Figure 5c). This suggests that a conductive fiber scaffold can provide efficient extracellular signaling pathways for regular and synchronized cell contraction. Reduced graphene oxide (rGO) functionalized electrospun silk nanofiber membrane (rGO/silk) endowed a significant elevation of V‐fib threshold (VFT) in infarcted rats, thus allowing the propagation of normal electrical signals.^[^ ^120^ ^]^

Melt electrospinning, as another mature fiber manufacturing technique, is widely used in the development of high‐performance functional fibers. This method enables large‐scale, automated production without the need for solvents. The process allows for tunable mechanical properties and thermal stability to meet diverse application requirements. By incorporating rGO into PVDF fibers, the crystallinity, thermal stability, and energy harvesting capabilities of the fibers can be significantly enhanced.^[^ ^121^ ^]^ The addition of rGO improves the crystallization dynamics of the fibers and increases their modulus and strength. In addition, PVDF/rGO nanocomposite fibers have been shown to effectively convert mechanical deformation into electrical signals while exhibiting good biocompatibility and cellular responsiveness (Figure 5d).^[^ ^121b^ ^]^ Notably, the helical structure of the fibers facilitates cell adhesion and growth under mechanical stimulation, making them suitable for use in pacemakers and other implantable medical devices.

Thanks to the convenience of ink modulation and the development of bioprinting, 3D printing can generally provide the precise realization of predefined functions in 3D space.^[^ ^122^ ^]^ As shown in Figure 5e, titanium carbide (Ti_3_C_2_T_x_) MXene was printed in a pre‐designed pattern on a polyethylene glycol (PEG) hydrogel, and the deposited nanosheets were restacked.^[^ ^122b^ ^]^ Representative curves of calcium fluxes of CMs on Ti_3_C_2_T_x_‐PEG composite hydrogels demonstrated a greater percentage of hopping cell area (82%) and a significant improvement in synchronized beating. Human pluripotent stem cell‐induced cardiomyocytes (iPSC‐CMs) and human umbilical vein endothelial cells (HUVECs) integrated in a hydrogel were printed to verify the effectiveness of electrical stimulation. The functional execution of CMs in unstimulated CM‐EC (CR) and stimulated CM‐EC (ES) was observed under 5.0 V cm^−1^ electrical stimulation conditions.^[^ ^123^ ^]^ Immunofluorescence staining showed that cardiomyocytes in the CM‐EC (ES) group formed ordered myofibroblasts on day 24. The results of similar frequencies of ROI time displacement curves and calcium transient curves both favorably confirmed that electrical stimulation significantly increased the contractile strength of CMs (Figure 5f). Mesenchymal stem cells (MSCs) can also be 3D printed to integrate biological and electrical signals. For example, 3D‐printed graphene oxide sheet (MSC@GO) hydrogel patches can be adherently attached to the epicardium to provide robust electrical integration to the infarcted heart and upregulate the expression of Cx43 to repair MI effectively.^[^ ^124^ ^]^ It is believed that the convenience of 3D bioprinting and the unlimited ability to program profiles can open up more and more possibilities for diagnosing and treating cardiac disease.

Advances in additive manufacturing technology give more possibilities for cardiac patch preparation methods. Carbon nanotube patches consisting of cellulose/single‐walled carbon nanotube inks by 3D printing on bacterial nanocellulose to integrate excellent electrical conductivity, flexibility, and stretchability could provide for improved cardiac conduction in the canine epicardium.^[^ ^125^ ^]^ 3D composite scaffolds constructed by encapsulating conductive nanofiber yarns in hydrogels can also mimic the complex interwoven structure of natural cardiac tissue and control cell alignment as well as induce the maturation of elongated CMs.^[^ ^126^ ^]^ The greatest advantage of composite scaffolds is that the unique properties of each component/preparation technique can be utilized to give the scaffolds stackable properties. As shown in Figure 5g, the nanocomposites using highly imprinted prepared Ag‐Au‐Pt core‐shell‐shell NWs (Ag‐Au‐Pt NWs) and in‐situ synthesized Pt NPs dispersed in styrene‐ethylene‐butadiene‐styrene (SEBS) block copolymers integrate the high electrical conductivity of Ag, the biocompatibility of Au, and the low impedance of Pt.^[^ ^127^ ^]^ Intrinsically soft mesh electrodes based on nanocomposites could be securely attached to the heart, and due to the effective charge transfer capability of the Ag‐Au‐Pt NW/Pt NP nanocomposites, upon stimulation at 0.75 V, the Ag‐Au‐Pt NW/Pt NP nanocomposite electrode has a minimum capture threshold of 0.67 V on average, which can induce and capture myocardial activity with much less energy. Meanwhile, 3D porous scaffolds can provide an environment for cardiomyocyte adhesion and facilitate improved electromechanical coupling of cardiomyocytes to host tissues.^[^ ^128^ ^]^ For example, the porous 3D scaffold composed of filipin and PPy significantly expresses cardiac marker proteins (𝜶‐actinin, Cx‐43, cTnT), which in vivo improve the propagation of electrical impulses, promote the synchronization of contraction of CMs in the scar region with the normal myocardium and effectively reduce the vulnerability of MI rats to cardiac arrhythmias.^[^ ^129^ ^]^ Integrating electronics with flexible substrates has also attracted the interest of researchers,^[^ ^130^ ^]^ an ideal bioelectronic device should have mechanical softness and deformability similar to cardiac tissue as well as a variety of sensing capabilities.^[^ ^131^ ^]^ Ag NW and SBS rubbers were chosen to prepare cardiac‐like tissue substrates that were effective in reducing stress, improving cardiac systolic function, and terminating ventricular tachycardia as an epicardial defibrillator.^[^ ^132^ ^]^ A pacemaker prepared by the fusion of flexible materials and electronic devices can perform overdrive/effective pacing to increase blood circulation in an anesthetized porcine model.^[^ ^133^ ^]^ Utilizing atomically thin electronic graphene with an equally transparent polymethylmethacrylate (PMMA) integrated bioelectronic interface, it allows for simultaneous cardiac action potential, calcium transient monitoring, and optogenetic therapy during electrical measurements and stimulation,^[^ ^134^ ^]^ as well as for successful restoration of ventricular rhythm during atrioventricular conduction block (Figure 5h). Some conductive hydrogels can also enhance device‐organ adhesion when integrated as a bioelectronic patch, enabling transient and compliant tissue adhesion on the heart for accurate cardiac monitoring.^[^ ^135^ ^]^ Yu et al. proposed a multifunctional adhesive hydrogel as an interfacial layer to be delicately integrated with a pacemaker for precise and continuous electrical stimulation to regulate cardiac rhythm to achieve stable and conformal bonding as well as stable electronic exchange between the pacemaker electrodes and the myocardium.^[^ ^136^ ^]^ Soft bioelectronic devices are being explored for use in a diverse range of therapeutic scenarios, and it is believed that they can play an increasingly important role in the high‐precision treatment of cardiac disorders under their excellent stretchability, flexibility, and a high degree of compatibility with biological organs and tissues.^[^ ^93^ ^]^

So far, an increasing range of techniques have been used to fabricate conductive scaffolds for cardiac repair. Some representative examples of fabrication techniques and electroactive materials are summarized in **Tables** **2** and **3**, respectively. While ensuring excellent electrical conductivity and matching tissue mechanical properties, there is an emerging trend to integrate soft electronic bio‐platforms for diagnostics and therapeutics or to combine with drug and cellular therapies for long‐term treatments.

**Table 2:** Representative types of conductive electroactive systems.

Fabrications	Substrate	Conductive filler	Conductivity [S cm^−1]^	Applications	Refs.
Hydrogel	GelMA	GNRs	7 × 10^−5^	Cardiac tissue engineering and maturation	[97b]
Hydrogel	ECM	MXene (Ti_3_C_2_)	5.29 × 10^−5^	Electrical integrity and signal propagation	[102]
Hydrogel	GelMA network interpenetrated with PGA	PDA‐hybridized PEDOT nanoparticles	>0.15	Myocardial repair and protection	[105]
Hydrogel	PVA+PAAm	PEDOT:PSS	9.3 × 10^−3^	Arrhythmia management	[108]
Hydrogel	Collagen	PEDOT:PSS	6.53 × 10^−2^	Arrhythmia management	[107]
Electrospinning	Polyethersulfone	PANI	5.7 × 10^−4^	Cardiac tissue engineering and maturation	[117b]
Electrospinning	PVA	PEDOT:PSS	7 × 10^−3^	Cardiac tissue engineering and maturation	[117c]
Electrospinning	Silk	rGO	0.2–0.3	Electrical integrity and signal propagation	[177c]
Melt spinning	PVDF	rGO	/	Arrhythmia management	[121b]
3D Printing	ECM hydrogel	Graphite flakes	0.3	Electrical integrity and signal propagation	[122a]
3D Printing	PEG	MXene (Ti_3_C_2_)	110	Cardiac tissue engineering and maturation	[122b]
3D Printing	Hydrophilic polyurethane	PEDOT:PSS	≈0.7	Arrhythmia management	[172]
In situ synthesis	Ag‐Au‐Pt nanowires	Pt NPs	11 × 10^3^	Electrical integrity and signal propagation	[127]
Salting‐out method	Silk fibroin	PPy	8 × 10^−3^	Myocardial repair and protection	[129]
Multilayer stacking	Sub‐micrometer‐thick, tissue‐conformable graphene arrays	Graphene	/	Electrical integrity and signal propagation	[134]

**Table 3:** Representative types of electroactive materials.

Types	Classifications	Electroactive substrates	Pros	Cons	Applications	Refs.
Triboelectric materials	Polymer‐Metals	PET/Au Kapton/Al n‐PTFE/Al	Self‐powered, high sensitivity,	Stability in wet environments, potential cytotoxicity	Energy harvesting, sensing, pacing, electrical stimulation	[65, 146, 161]
	Polymers	PVA‐NH_2_/PFA	Self‐powered, biocompatible, environmentally‐ friendly	Limited output, sensitivity to humidity		[166]
Piezoelectric materials	Inorganic materials	BaTiO_3_/ZnO	Self‐powered, high piezoelectric coefficient	Potential cytotoxicity, lack of flexibility, processing complexity	Energy harvesting, sensing, neuromodulation	[59, 82]
	Organic materials	PVDF/P(VDF‐TrFE) /PLLA/PHBV	Flexibility, biodegradable(partial)	Weaker output, lower durability		[60, 61, 165]
Conductive materials	Metals	Au/Al/Cu/Ag	High conductivity	Poor biocompatibility, corrosion	Monitoring, repairing, and signal synchronization	[66, 68, 141, 146]
	2D materials	MXene/rGO	High surface area, versatile properties	Cost of synthesis, stability		[99, 100, 102]
	Polymers	PEDOT:PSS/PPy/PANI/ PAMB	Biocompatibility, tunable elasticity	Long‐term mechanical stability		[103, 104, 107, 109]
Composite materials	/	Ti_3_C_2_T_x_‐PEG/ Ag‐Au‐Pt NWs/ Graphene‐PMMA	Combines electrical and mechanical benefits	Complexity in fabrication	Multi‐function	[122, 127, 134]

## 4 Applications in Cardiac Arrhythmia

The applications of electroactive platforms in cardiac arrhythmias are diverse and hold significant promise for enhancing patient outcomes. This chapter explores the multifaceted roles these platforms play, focusing on their contributions to arrhythmic episode monitoring, therapeutic interventions, innovative pacing strategies, and the restoration of electrical integrity, thereby emphasizing their potential to revolutionize the management of cardiac arrhythmias.

### 4.1 Monitoring‐Enhancing Surveillance

Early diagnosis and timely intervention are critical steps in improving outcomes in the management of arrhythmias.^[^ ^137^ ^]^ Traditional electrocardiography (ECG) records the heart's electrical activity by placing electrodes at specific locations on the patient's body. These electrodes can detect the weak electrical signals generated by the depolarization and repolarization processes of myocardial cells, which are then amplified and converted into visual waveforms presented on paper or digital displays.^[^ ^138^ ^]^ Despite its widespread use, ECG has notable limitations. Due to device and power constraints, ECG typically provides intermittent measurements, making continuous, real‐time monitoring of cardiac activity challenging and often missing transient arrhythmic episodes such as subclinical AF. Moreover, the weak signals collected from the body surface are susceptible to interference, impacting diagnostic accuracy. Conventional ECG devices also lack remote transmission and real‐time analysis capabilities. Although the advent of dynamic electrocardiography (Holter) has partially alleviated these issues, its size and bulk still limit its application in telemedicine and continuous monitoring.^[^ ^139^ ^]^

**Figure 6:**
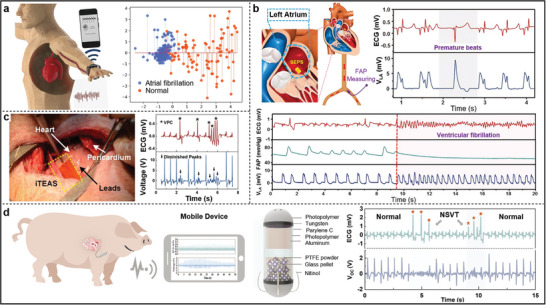
a) Information transmission and LDA cluster distribution of the AFPW. Reproduced with permission.^[^ ^60^ ^]^ Copyright 2023, Springer Nature. b) Schematic and ECG signals showing SEPS implantation (upper left) and its response to VPC (upper right) and VF (lower). Reproduced with permission.^[^ ^143^ ^]^ Copyright 2018, Wiley‐VCH. c) Implantation of the iTEAS between the pericardium and epicardium, and its detection of ventricular premature contractions. Reproduced with permission.^[^ ^65^ ^]^ Copyright 2016, American Chemical Society. d) Schematic diagram of signal processing and transmission in vivo experiment, structure of the BCMC, and disorganized signal waveforms during NSVT. Reproduced with permission.^[^ ^146^ ^]^ Copyright 2024, Wiley‐VCH.

In the context of advancements in flexible technology, big data, and artificial intelligence, wearable sensors have shown significant potential for monitoring and diagnosing arrhythmias.^[^ ^63^ ^]^ These sensors are often integrated into form‐fitting clothing or portable devices, allowing for non‐invasive, continuous monitoring through the detection of pulse information.^[^ ^140^ ^]^ As shown in **Figure** **6**a, the piezoelectric‐based atrial fibrillation prediction wristband (AFPW) capitalizes on irregularities in pulse waves during AF to facilitate early predictions.^[^ ^60^ ^]^ Testing at various frequencies demonstrates the AFPW's excellent adaptability during rapid atrial contractions. Additionally, a machine‐learning‐based AI model was developed using a clinical sample database, where features extracted from different pulse wave signals were analyzed using a linear discriminant analysis (LDA) model, achieving a diagnostic accuracy of up to 91%. Similarly, another self‐powered ultrasensitive pulse sensor (SUPS), which operates on a triboelectric active sensing mechanism, offers non‐invasive monitoring when worn on the finger or wrist.^[^ ^141^ ^]^ This sensor captures voltage signals corresponding to the reciprocal of the pulse signal. Constructed from nanostructured Kapton (n‐Kapton) film using ICP etching, it significantly enhances output. Both technologies transmit data wirelessly to mobile devices via Bluetooth and can upload information to cloud servers for healthcare professionals to access, enabling remote monitoring of AF.

As challenges related to biocompatibility, miniaturization, and long‐term stability are addressed, implantable cardiac monitoring devices are gaining attention. Compared to wearable devices, implantable devices can be securely placed within the body, and positioned directly in or near the heart or blood vessels. This proximity allows for more precise capture of electrocardiographic signals, significantly improving signal sensitivity and effectively reducing external interference.^[^ ^142^ ^]^ The iTEAS can be sutured to the pericardium in a minimally invasive manner, directly sensing cardiac contractions to achieve accurate heart rate monitoring.^[^ ^65^ ^]^ Each heartbeat creates rapid contact and separation between the heart and the iTEAS device, generating electrical signals transmitted through an external multi‐channel data acquisition (DAQ) system. To validate the device's capability for arrhythmia monitoring, the researchers employed an aconitine‐induced AF pig model. The experimental results showed a drop in output voltage with varying frequency, perfectly mirroring the irregular p‐p intervals associated with AF. Similarly, during induced ventricular premature contractions (VPC), the iTEAS was able to accurately capture abnormal cardiac rhythm changes, with output voltage signals displaying similar characteristic variations (Figure 6b).

Sensors implanted within cardiac chambers can directly sense the impact of each heartbeat on blood flow, yielding more biomedical information. For instance, the self‐powered endocardial pressure sensor (SEPS), shown in Figure 6c, is inserted into the left ventricle or left atrium via a catheter.^[^ ^143^ ^]^ Based on the TENG principle, the blood flow within the cardiac chambers causes relative motion between the SEPS's two friction layers, generating electrical signals whose frequency and amplitude are directly related to intracardiac pressure (ICP). Elevated ICP is associated with increased risks of VT and VF.^[^ ^144^ ^]^ Therefore, SEPS can monitor abnormal arrhythmic events by capturing sudden changes in ICP. In experimental pigs, the changes in VPC and Voc induced by a temporary pacemaker corresponded well, while during VF, the Voc lost the regular waveform it exhibited under sinus rhythm and became erratic in both rhythm and amplitude. In addition to ICP, myocardial contractility also changes during arrhythmic events.^[^ ^145^ ^]^ Inspired by this, Qu et al. developed a fully implanted bias‐free cardiac monitoring capsule (BCMC), which is implanted via the subclavian vein into the right ventricular apex.^[^ ^146^ ^]^ The capsule is securely anchored with four nickel hooks between the papillary muscles and connects to an under‐skin data acquisition and transmission module for real‐time signal monitoring (Figure 6d). The capsule's inner wall is coated with aluminum foil, and it contains glass pellets with PTFE powder. When the glass pellets move relative to the aluminum foil, the charge distribution changes, generating electrical signals. The BCMC responds rapidly under conditions of increased myocardial contractility induced by dopamine and aligns well with changes in femoral artery pressure (FAP) signals. While during PVC and non‐sustained ventricular tachycardia (NSVT), the localized myocardial contractility decreased, and the BCMC signal correspondingly declined. This highly sensitive, high SNR (42 dB) implanted capsule shows significant advantages in arrhythmia monitoring, paving the way for innovative clinical applications.

Electroactive systems have revolutionized arrhythmia monitoring by enabling the detection of transient episodes which are often missed by traditional devices, such as subclinical AF and VPC. Wearable sensors provide non‐invasive, real‐time data collection during daily activities, while implantable systems enable precise, long‐term monitoring with superior signal fidelity. These advancements mark a critical step toward more efficient and accessible cardiac monitoring technologies.

### 4.2 Intervention‐Advancing Treatment

Building upon the exploration of diverse applications of electroactive materials in cardiac therapy, this section delves into their advanced roles in managing arrhythmias. By leveraging their unique conductive and mechanical properties, these materials have shown promise in addressing specific challenges posed by arrhythmias, such as atrial fibrillation (AF) and ventricular arrhythmias, through mechanisms that restore electrical conduction and structural integrity within cardiac tissues.

**Figure 7:**
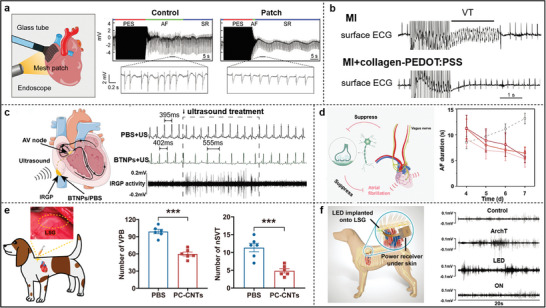
a) Illustration of the injectable mesh‐like conductive hydrogel patch implantation process using an endoscope, and the corresponding ECG showing the elimination of AF after patch application. Reproduced with permission.^[^ ^108^ ^]^ Copyright 2024, Wiley‐VCH. b) Surface ECG showing the reduction of VT incidence in MI mouse hearts treated with collagen‐PEDOT:PSS hydrogel compared to untreated hearts. Reproduced with permission.^[^ ^153^ ^]^ Copyright 2024, Wiley‐VCH. c) Schematic illustration of the ultrasound activation of the piezoelectric nanoparticles in the IRGP and representative IRGP activity changes in the BTNP group after ultrasound treatment. Reproduced with permission.^[^ ^82^ ^]^ Copyright 2023, The Royal Society of Chemistry. d) Illustration of the self‐powered LL‐VNS system and the comparison of AF duration between the model group and the experimental group treated with LL‐VNS. Reproduced with permission.^[^ ^68^ ^]^ Copyright 2022, Elsevier. e) Anatomical location of the LSG, and the effects of PC‐CNTs on the reduction of ventricular premature beats (VPBs) and NSVT incidence. Reproduced with permission.^[^ ^160^ ^]^ Copyright 2023, American Chemical Society. f) Schematic diagram of the wireless self‐powered optogenetic system implantation and its effects on LSG function. Reproduced with permission.^[^ ^161^ ^]^ Copyright 2023, Wiley‐VCH.

AF is characterized by rapid and chaotic atrial contractions, resulting in irregular ventricular contractions and decreased effective cardiac output.^[^ ^147^ ^]^ This irregular electrical activity disrupts the normal rhythm of the heart, increasing the risk of thrombus formation, which can lead to stroke or other embolic events.^[^ ^148^ ^]^ Epicardial patches with good conductivity can effectively guide and synchronize the conduction of atrial electrical signals, thereby improving rhythm stability. Examples include a flexible biocompatible PPy‐PCNU composite film with semiconductor properties^[^ ^117a^ ^]^ and a PAMB‐G hydrogel that closely mimics cardiac mechanical properties.^[^ ^149^ ^]^ These designs optimize the conduction characteristics of atrial electrical signals, helping to overcome local conduction barriers caused by cardiac fibrosis and reducing the duration of AF. On this basis, an injectable mesh conductive hydrogel patch based on PVA, PAAm, and PEDOT: PSS was fabricated, which endows a unique diamond structure (**Figure** **7**a).^[^ ^108^ ^]^ This patch not only exhibits excellent conductivity and high charge storage capacity but also demonstrates superior mechanical elasticity and shape memory characteristics. After endoscopic implantation in the right atrium, it closely adheres to the cardiac surface and adapts to deformations during the cardiac cycle, significantly reducing the duration of AF and effectively restoring sinus rhythm.

In addition, ventricular arrhythmias are often closely related to myocardial infarction (MI). Coronary artery blockage leads to myocardial ischemia, resulting in the loss of a significant number of cardiomyocytes, which exceeds the heart's regenerative capacity.^[^ ^150^ ^]^ This is followed by repair events characterized by sterile inflammation and immune cell infiltration, ultimately resulting in fibrotic scar tissue.^[^ ^151^ ^]^ The electrical conductivity of fibrotic tissue is notably poor, and interactions with the heterocellular border region contribute to abnormal electrical activity in the ventricles. This disruption results in rapid and disorganized contractions of the ventricles, which significantly increases the risk of SCD.^[^ ^152^ ^]^ Another conductive hydrogel based on PEDOT: PSS further confirms the application value of this material in ventricular arrhythmias.^[^ ^153^ ^]^ The combination of collagen with PEDOT: PSS not only enhances the formation and micro‐morphology of collagen gels but also significantly improves their conductivity, facilitating electrical conduction. After transplanting hiPSC‐derived cardiomyocytes, the damaged myocardial tissue in the affected area can be further restored, reducing the levels of VT (Figure 7b).

As previously mentioned, ANS plays an irreplaceable role in regulating heart rhythm, and abnormal activity of the ANS is also a significant cause of arrhythmias.^[^ ^154^ ^]^ Through a deeper understanding of the neuroanatomical circuits at the cardiac level, new approaches to treating arrhythmias have emerged,^[^ ^29^ ^]^ including neuromodulation,^[^ ^155^ ^]^ surgical resection or ablation of specific ganglia,^[^ ^156^ ^]^ and gene therapy targeting cardiac neural effector sites.^[^ ^157^ ^]^ Related studies combine the design of electroactive materials with innovative devices to achieve effective ANS modulation. The LL‐VNS system,^[^ ^68^ ^]^ based on triboelectric and piezoelectric effects, harnesses energy from pulsations and automatically activates VNS upon detecting the occurrence of AF, thereby reducing its duration and improving associated pathological changes (Figure 7c). Controlling ventricular rate during AF to ensure effective blood supply is another vital therapeutic strategy.^[^ ^158^ ^]^ Han et al. implanted barium titanate nanoparticles (BTNPs) into the inferior right ganglionated plexus (IRGP), and observed significant changes in neural activity after activation using external ultrasound equipment (Figure 7d).^[^ ^82^ ^]^ This approach successfully controlled ventricular rate during AF. The left stellate ganglion (LSG) also represents an important target for neuromodulation; its inhibition can improve ventricular arrhythmia.^[^ ^159^ ^]^ Xu et al. developed a novel neuromodulator PC‐CNTs by utilizing the excellent electrical conductivity of CNTs and their strong interfacing capability with neurons, along with the biocompatibility of phospholipids.^[^ ^160^ ^]^ Following injection into the LSG, it was shown to inhibit its discharge frequency and reduce neuroinflammatory responses (Figure 7e), thereby mitigating ventricular arrhythmia after MI. In addition, light stimulation has an important role in cardiac neural regulation.^[^ ^161^ ^]^ By targeting the LSG neurons to express the inhibitory light‐sensitive protein ArchT gene and placing an implanted LED device nearby which was powered by a TENG. This self‐powered system achieved long‐term sympathetic nerve modulation, significantly alleviating cardiac remodeling and malignant arrhythmias after MI, providing preliminary validation for the potential of optical stimulation in long‐term modulation of LSG activity (Figure 7f).

Advances in electroactive materials have propelled arrhythmia treatment into new territories, leveraging conductive patches and hydrogels to restore disrupted electrical pathways in conditions like post‐MI arrhythmias. Meanwhile, neuroregulatory innovations enhance autonomic control, offering a multifaceted approach to rhythm control. These diverse approaches highlight the growing impact of adaptive materials in addressing arrhythmias.

### 4.3 Pacing‐Revolutionizing Modalities

**Figure 8:**
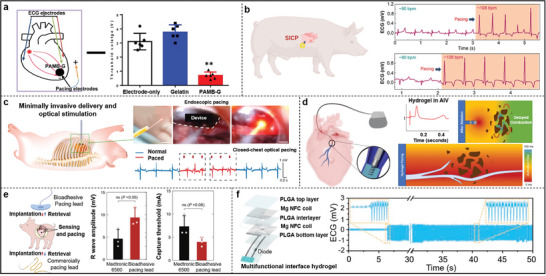
a) Schematic illustration of the Langendorff‐perfused rat heart pacing model with the injectable conductive hydrogel (PAMB‐G) and comparison of pacing threshold voltages, showing that PAMB‐G significantly reduces the pacing threshold compared to electrode‐only and. Reproduced with permission.^[^ ^163^ ^]^ Copyright 2021, Open Access. b) Schematic diagram of SICP working in vivo and ECG signals at 3 weeks after implantation showing normal pacing function. Reproduced with permission.^[^ ^67^ ^]^ Copyright 2024, Springer Nature. c) Schematic of the photostimulation setup and representative ECG waveforms showing the pacing of a live pig heart using optical pulses in a fully closed‐chest procedure. Scale bars, 1 cm (left), 5 mm (shared by middle and right). Reproduced with permission.^[^ ^89^ ^]^ Copyright 2024, Springer Nature. d) Schematic of the hydrogel electrode connection to a pacemaker lead, corresponding ECG of pacing from hydrogel in the AIV, and electroanatomical mapping showing earlier and more uniform activation with hydrogel pacing in an ablation model. Reproduced with permission.^[^ ^171^ ^]^ Copyright 2024, Springer Nature. e) Schematic of the bioadhesive pacing lead implantation and successful ventricular pacing with high R wave amplitude and low capture threshold. Reproduced with permission.^[^ ^172^ ^]^ Copyright 2024, The American Association for the Advancement of Science. f) Schematic of the structure of MIH and representative ECG waveforms showing successful pacing with stable R wave amplitudes in a rat model. Reproduced with permission.^[^ ^173^ ^]^ Copyright 2024, Elsevier.

In 1958, Swedish physician Rune Elmqvist and cardiac surgeon Åke Senning jointly developed the world's first fully implantable cardiac pacemaker, marking a significant milestone in the collaboration between medicine and engineering.^[^ ^162^ ^]^ Over time, this interdisciplinary partnership has deepened, driving continuous advancements in pacemaker technology. The progress of electroactive materials has played a crucial role in optimizing traditional pacemaker technology, particularly with the development of conductive hydrogels, conductive polymers, and nanocomposites, which offer new possibilities for improving the electrode‐tissue interface. Recent research highlights the potential of an injectable conductive hydrogel in optimizing pacemaker performance.^[^ ^163^ ^]^ Evaluation through a Langendorff‐perfused heart model demonstrated that this PAMB‐G hydrogel significantly reduced the pacing threshold and improved pacing efficiency while enhancing the conduction velocity (**Figure** **8**a). The application of the hydrogel not only promises to decrease the energy consumption of pacemakers but also may mitigate adverse effects such as localized fibrosis associated with long‐term use, providing insights into addressing issues of battery depletion and increased impedance of traditional pacemakers. Moreover, a novel anti‐fatigue conductive polymer hydrogel coating presents a fresh approach to the durability of implantable bioelectronic devices.^[^ ^164^ ^]^ This hydrogel, composed of PVA and PEDOT: PSS, successfully achieves an interface fatigue threshold of up to 330 J m^−2^ by constructing nanocrystal domains between it and the metal substrate, demonstrating exceptional anti‐fatigue properties. It not only effectively reduces the pacing threshold voltage of pacemakers but also significantly enhances the long‐term reliability of electrical stimulation. In vitro and in vivo experiments further validated its biocompatibility and long‐term stability. In the quest for bioelectronic interface materials, alongside the previously mentioned conductive hydrogel coatings, another groundbreaking study focused on developing stretchable low‐impedance conductors. Sunwoo et al. proposed an innovative material design using silver‐gold‐platinum (Ag‐Au‐Pt) core‐shell nanowires with in situ‐formed platinum nanoparticles.^[^ ^127^ ^]^ This novel stretchable conductor shows great promise for applications in wearable and implantable bioelectronic devices with lower pacing thresholds, higher signal quality, and longer lifespans.

Meanwhile, emerging energy harvesting technologies offer innovative solutions for the long‐term power supply of pacemakers, redefining energy management strategies for CIEDs. The use of the body's intrinsic bioenergy to convert into electrical energy, particularly through non‐invasive energy harvesting NG technologies, offers distinct advantages in this regard. In the realm of PENG, an implantable nanogenerator (i‐NG) utilizing gold‐coated PVDF as the core material has demonstrated the feasibility of generating electrical energy from cardiac motion.^[^ ^165^ ^]^ This device is implanted in the epicardium, where the deformation of the PVDF film during cardiac contraction and relaxation generates electrical output. Two months of animal experiments revealed sustained energy generation capacity and good biocompatibility. A further TENG‐based symbiotic cardiac pacemaker (SPM), also implanted in the epicardium, has undergone design optimizations that greatly enhance the device's energy‐harvesting efficiency.^[^ ^66^ ^]^ The energy harvested from heartbeats by the iTENG can be stored in the power management unit (PMU), enabling pacing functionality after activation by an external wireless passive trigger. In vivo animal experiments demonstrated that under normal heart rate and blood pressure conditions, the SPM could charge to ≈4 V within 63 min which is capable of correcting sinus arrhythmia and restoring heart rate and blood pressure. Subsequent studies have further advanced miniaturization and efficiency improvements, such as the fully implantable SICP shown in Figure 8b.^[^ ^67^ ^]^ While maintaining high efficiency, the SICP has been miniaturized to a capsule shape with a volume of only 1.52 cubic centimeters and a weight of just 1.75 grams. This miniaturization enables it to be delivered directly through a catheter and implanted at the right ventricular apex, significantly reducing interference from surrounding tissues. Under the influence of right ventricular blood flow, the POM particles inside the capsule come into contact with and separate from the PTFE film to generate electrical energy, demonstrating sustained pacing ability over three weeks in animal experiments. In addition to harnessing energy from heartbeats, the human body presents other potential energy sources.^[^ ^62^ ^]^ Ryu et al. developed an inertial‐driven triboelectric nanogenerator (I‐TENG) the size of a coin battery.^[^ ^166^ ^]^ The I‐TENG effectively converts vertical bodily movements into electrical energy. Even minor movements during rest or sleep can charge the I‐TENG, which demonstrated an energy conversion efficiency of up to 4.9 µW cm^−^^3^ in laboratory tests.

In pursuit of more efficient and reliable energy harvesting methods, the photovoltaic effect has emerged as another promising research direction. Unlike nanogenerators that rely on mechanical motion, the photovoltaic effect generates electrical current through the interaction of photons with materials (such as silicon‐based materials) within biological tissues, offering a novel energy supply pathway for CIEDs.^[^ ^167^ ^]^ For instance, researchers have developed a heart pacemaker based on flexible and biodegradable amorphous silicon (a‐Si:H) radial junction photovoltaic devices, successfully driving cardiac pacing in small pigs using a 650 nm light source.^[^ ^168^ ^]^ A further development involves a spatiotemporal photostimulation system consisting of single‐crystal silicon (sPN‐Si) and PIN‐Si structures.^[^ ^89^, ^169^ ^]^ These photovoltaic conversion materials enable precise control of the spatial and temporal distribution of photocurrent by finely tuning their nanostructure and doping distribution, achieving significant advancements in performance metrics: a photovoltaic conversion efficiency of 18.4%, temporal resolution improved to sub‐millisecond (<1 ms), and spatial resolution reaching micrometer scales (<10 µm). This high‐precision optical stimulation not only allows for precise heart rate modulation but also facilitates control of complex cardiac electrophysiological activities, successfully achieving light‐mediated cardiac pacing in large animal models through minimally invasive surgeries (Figure 8c). Notably, the device's weight is only one‐tenth that of traditional pacemakers, and this significant miniaturization is expected to greatly enhance patient comfort and quality of life. These advances collectively propel optical cardiac pacing technology closer to clinical application while providing crucial technical foundations and theoretical support for future research in related fields.

Finally, researchers are redefining the interaction between cardiac tissue and electronic devices by developing innovative bioelectronic interfaces. Hydrogels are being explored as potential flexible electrodes due to their tunable mechanical and chemical properties, as well as their ease of incorporation of conductive elements such as nanoparticles or conductive polymers.^[^ ^170^ ^]^ This adaptability allows for precise control over their stiffness and conductivity, thereby better meeting the mechanical and electrical demands of dynamic biological tissues such as the heart. As shown in Figure 8d, a new type of PEUDAm hydrogel has been developed by introducing hydrolysis‐resistant carbamate and amide groups into the structure of PEG.^[^ ^171^ ^]^ This hydrogel exhibits excellent biocompatibility, mechanical properties, and enhanced stability, making it suitable for the creation of flexible electrodes for vascular implantation. By injecting the hydrogel into the coronary veins and tributaries, pacing can be achieved across myocardial infarction areas caused by arterial blockage. This method helps normalize the activation of heterogeneous myocardial tissues and reduce delayed conduction. Notably, the hydrogel can achieve uniform capture along the vessel, mimicking the multi‐site pacing effect of multipolar catheters. In addition, bioadhesive materials offer new possibilities for pacing, exemplified by the bioadhesive pacing lead developed by Deng et al. (Figure 8e).^[^ ^172^ ^]^ Unlike traditional leads that require surgical suturing or direct insertion into cardiac tissue, these leads are made from 3D‐printable polyurethane‐based bioadhesive materials, optimized through grafting of polyacrylic acid and blending with PEDOT to achieve a balance between mechanical performance and conductivity. Utilizing physical and covalent interactions, they form a stable electrical interface with cardiac tissue. After two weeks of stable continuous pacing, the bioadhesive leads were removed in animal models by infusing a separating solution, and no significant tissue damage was observed. This design effectively reduces the trauma associated with the implantation and removal of traditional leads. A multifunctional interface hydrogel (MIH) also provides new insights into innovations in this area.^[^ ^173^ ^]^ As shown in Figure 8f, the MIH is composed of Ag NPs, gallic acid (GA), gelatin, and a double‐network hydrogel formed from polyacrylic acid (PAA) and potassium 3‐sulfopropyl methacrylate (MASPS). As a bioelectronic‐tissue interface material, the MIH exhibits excellent electrical exchange properties and bioactivity, enabling efficient electrical stimulation. MIH also features biodegradability, which demonstrates great potential for wireless transient pacemakers, reducing surgical complications during device implantation and removal.

Emerging electroactive platforms have addressed long‐standing challenges in cardiac pacing. From conductive hydrogels that optimize electrode‐tissue interfaces to energy harvesting systems like TENG and PENG that sustain device power, these technologies provide transformative solutions. Bioadhesive interfaces further enhance pacing reliability and minimize trauma. Collectively, these technologies advance cardiac pacing by addressing limitations in traditional designs and aligning with evolving clinical demands for reliability and patient‐centered care.

### 4.4 Repairing‐Restoring Electrical Integrity

The normal rhythm of the heart depends on effective electrical signal transmission among cardiomyocytes via gap junctions. When MI occurs, the cells in the affected region undergo necrosis, resulting in localized tissue scarring, which disrupts normal conduction pathways and creates electrically isolated regions or ectopic pacemakers, thus resulting in arrhythmias.^[^ ^174^ ^]^ Restoring the electrical conduction properties of damaged myocardium through innovative therapeutic strategies enhances cardiac pump function and reduces the risk of arrhythmias linked to conduction abnormalities. These strategies encompass gene therapy,^[^ ^175^ ^]^ cell therapy,^[^ ^176^ ^]^ and the application of conductive biomaterials^[^ ^177^ ^]^ to enhance electrical coupling between implanted cells or materials and the host myocardium, thereby reconstructing normal excitation conduction pathways and ultimately optimizing cardiac electrical activity.

**Figure 9:**
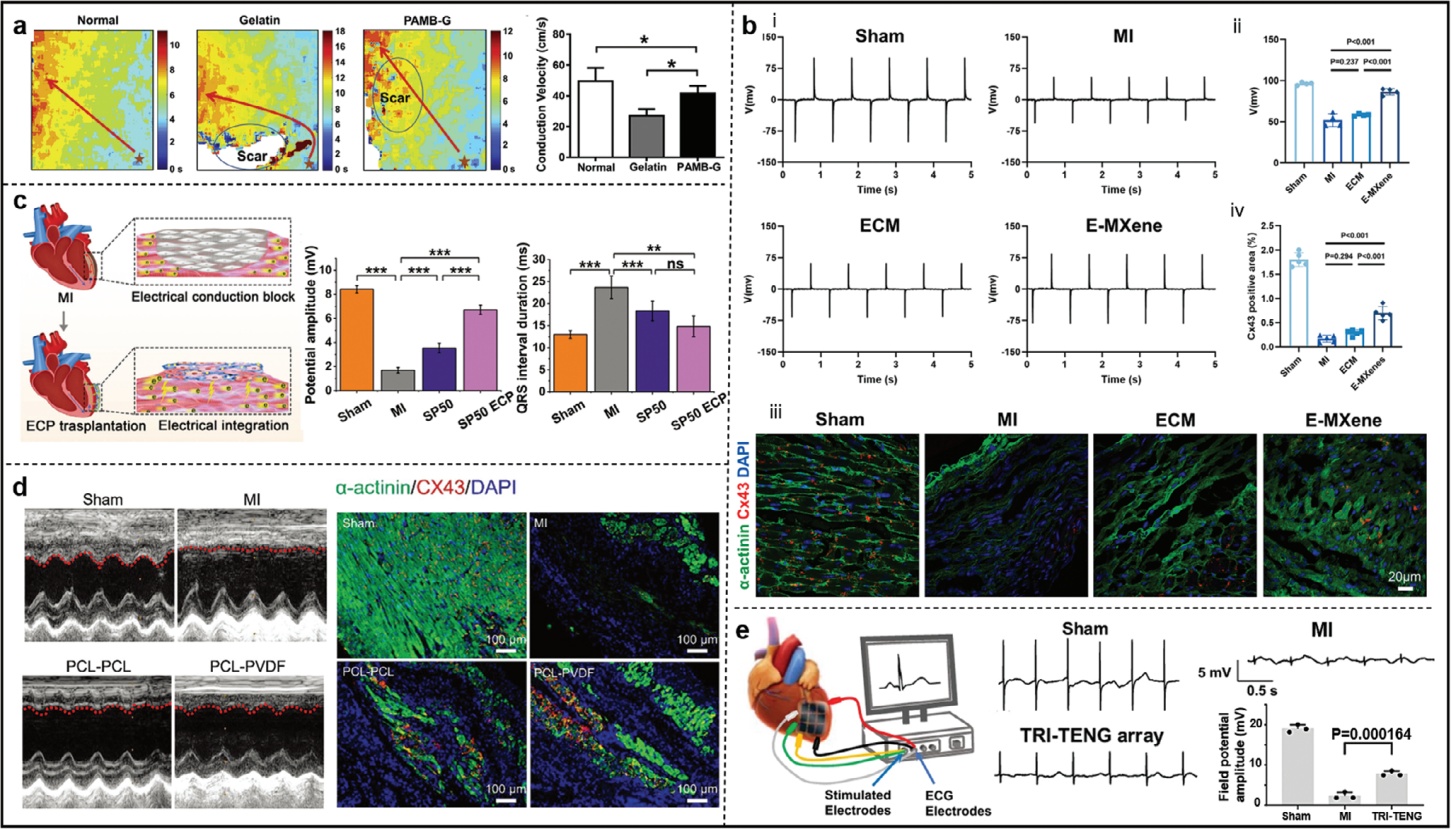
a) Optical mappings showing improved electrical impulse propagation. Reproduced with permission.^[^ ^178^ ^]^ Copyright 2020, Elsevier. b) The effect of E‐MXene hydrogel on interstitial electrical conductance in MI tissue, showing i) a schematic of detecting electrical signals through the infarction area, ii) quantitative data of electrical signal amplitudes, iii) immunofluorescence staining of Cx43 and α‐actinin 28 days post‐MI, and iv) statistics of the positive fluorescence area of Cx43 in the MI region. Reproduced with permission.^[^ ^180^ ^]^ Copyright 2024, Elsevier. c) Schematic illustration of the application SP50ECP in MI repair, and improved local field potential amplitudes and QRSd in the infarcted myocardium. Reproduced with permission.^[^ ^185^ ^]^ Copyright 2023, Wiley‐VCH. d) Representative M‐mode parasternal long‐axis view of LV echocardiographic images and staining images of 𝛼‐actinin and CX43 in the infarcted area in different groups. Reproduced with permission.^[^ ^78^ ^]^ Copyright 2024, Wiley‐VCH. e) Representative ECG traces demonstrating the restoration of electrical conduction following TRI‐TENG array transplantation. Reproduced with permission.^[^ ^186^ ^]^ Copyright 2024, Springer Nature.

Conductive hydrogels, with their superior biocompatibility and conductivity, can bridge damaged cardiomyocytes, effectively restoring electrical conduction. As illustrated in **Figure** **9**a, direct injection of the PAMB‐G hydrogel into the infarcted area results in increased amplitude of current propagation and faster conduction velocities, with two‐dimensional mapping revealing pathways more akin to normal myocardium.^[^ ^178^ ^]^ Subsequent long‐term studies confirm the biocompatibility, and durability over one year.^[^ ^179^ ^]^ Incorporating functional additives into conductive biomaterials can further promote myocardial repair and stimulate tissue regeneration while restoring electrical conduction. For instance, the addition of MXene nanosheets (Ti_3_C_2_) to thermoresponsive extracellular matrix (ECM) hydrogels enhances calcium signaling synchronization and reduces reactive oxygen species (ROS) levels (Figure 9b).^[^ ^180^ ^]^ PAMB can also be grafted with carboxymethyl chitosan and encapsulated with human umbilical mesenchymal stem cell secretome, yielding hydrogels that not only exhibit excellent electrical conductivity but also enhance angiogenesis and mitigate inflammation through growth factors and extracellular vesicles present in the secretome.^[^ ^181^ ^]^ Similarly, exosomes derived from human endometrial mesenchymal stem cells (hEMSC‐Exo) were integrated into PPy‐chitosan composites.^[^ ^182^ ^]^ During hydrogel degradation, these exosomes are gradually released, exerting long‐term therapeutic effects. They promote angiogenesis and inhibit apoptosis via the EGF/PI3K/AKT signaling pathway, thus mitigating wall remodeling and fibrosis.

Pre‐seeding cardiomyocytes onto conductive scaffolds enhances their electrical coupling and calcium handling, facilitating mechanical and electrical integration with the host myocardium and potentially improving functional recovery.^[^ ^183^ ^]^ For example, a 3D porous structure formed by combining PAMB with gelatin‐based foam simulates the microenvironment conducive to cardiomyocyte growth and maturation, thus enhancing local electrical conduction.^[^ ^184^ ^]^ Similarly, Yin et al. developed an electrically active engineered cardiac patch (SP50 ECP) from a sponge‐like porous conductive scaffold created using silk fibroin and PPy.^[^ ^185^ ^]^ By seeding cardiomyocytes onto this scaffold and applying electrical stimulation, they promoted the maturation of these cells. (Figure 9c). This approach improved QRS duration (QRSd) after MI, facilitated vascular integration, and restored electrical connectivity in the infarcted area.

As noted earlier, mechanical synergistic electrical stimulation promotes cardiomyocyte maturation and normal rhythmic contraction. Remarkably, the magnetoelectric effect has also shown significant potential. A cardiac patch with serpentine microstructures can provide physiologically relevant electrical stimulation utilizing magnetoelectric coupling, enhancing cardiomyocyte maturation and intercellular connectivity (Figure 9d).^[^ ^78^ ^]^ Moreover, a self‐powered tri‐functional triboelectric nanogenerator (TRI‐TENG) cardiac patch was fabricated for the integration of multiple functions, featuring a unique dual‐spacer structure comprising Ecoflex film, reduced rGO electrodes, PVDF film with a venation structure, and PDA‐modified rGO electrodes (Figure 9e).^[^ ^186^ ^]^ This design harnesses cardiac mechanical motion to generate electrical stimulation through friction at the PDA‐rGO interface to reduce the risk of malignant arrhythmias following MI. The TRI‐TENG integrates energy harvesting, therapeutic intervention, and remote diagnostics, presenting a novel approach for restoring cardiac rhythm post‐myocardial infarction.

Electroactive materials, including hydrogels and engineered cardiac patches, present versatile solutions for repairing myocardial conduction deficits. Hydrogels bridge damaged regions and promote tissue regeneration, while systems like TRI‐TENG integrate therapeutic stimulation with energy harvesting. These advances demonstrate the versatility of emerging materials in addressing both localized damage and broader conduction deficits.

## 5 Conclusions and Perspectives

In this review, we comprehensively summarize various studies of electroactive platforms applied to the management of cardiac arrhythmias. We categorize electroactive platforms into four types based on their origin: i) Direct electrical stimulation, ii) Self‐powered electroactive systems, iii) Physical stimuli‐mediated electroactive systems, and iv) Conductive systems. We introduce their applications in the arrhythmia field, encompassing monitoring, intervention, pacing, and repairing. Overall, the advancements in these technologies are promising, offering new perspectives for future arrhythmia treatments. They have the potential to revolutionize existing therapies and establish a robust foundation for the broader application of electroactive platforms. Despite the high potential of applying electroactive technologies in arrhythmia management, several challenges still remain.

### 5.1 Flexibility and Biocompatibility

Given the heart's location, achieving optimal functionality for electroactive platforms often necessitates open‐heart surgery for direct implantation or interventional procedures to implant them in proximity to the heart. This requires stringent compliance with flexibility and biocompatibility standards. Moreover, devices implanted within the cardiac cavity or blood vessels demand exceptionally high blood compatibility to mitigate thrombotic risks.^[^ ^187^ ^]^ Currently, most biocompatibility assessments are conducted under ideal laboratory conditions or in animal models, which cannot fully replicate the complexities of the human bodily fluid environment, leaving long‐term testing relatively unexplored. On the other hand, once implanted, encapsulation materials face additional challenges, such as degradation in humid environments, which can impair their mechanical and electrical properties. Therefore, a series of criteria need to be considered in encapsulation material design, including flexibility, blood compatibility, and bioadhesion, to ensure long‐term stability.

### 5.2 Adaptability and Immunity

Furthermore, optimizing the adaptation and integration of these materials with tissue interfaces to achieve better electrical conduction is a significant research focus. The heart's irregular surfaces and chambers, along with its continuous dynamic motion, make it challenging for seamless integration. This instability at the interface may lead to signal transmission interruptions and foreign body reactions.^[^ ^188^ ^]^ Advances in bioelectronic interface technology hold promise for overcoming this limitation. By employing more sophisticated materials and design strategies,^[^ ^189^ ^]^ including conductive bioadhesives,^[^ ^190^ ^]^ intrinsically stretchable electronic materials with low Young's modulus,^[^ ^191^ ^]^ and water‐responsive supercontracting polymer films,^[^ ^192^ ^]^ these technologies may improve interface flexibility and adaptability in the future, thus enhancing the integration of materials with cardiac tissue. In addition, the interaction between electroactive materials and the immune system requires further investigation to minimize immune responses and ensure the long‐term safety and effectiveness of implanted devices.

### 5.3 Miniaturization and High Efficiency

Breakthroughs in miniaturization and integration of electroactive systems will significantly enhance their application in implantable devices, so it is essential to consider the design of miniaturized devices and the development of non‐invasive therapeutic solutions. In the current clinical setting, the use of CIEDs relies on external power supplies. A subcutaneous pocket is typically sewn into the patient's chest to accommodate the pulse generator, causing considerable discomfort and a risk of infection that severely affects prognosis.^[^ ^193^ ^]^ Self‐powered technologies, primarily based on NG technology, offer new hope for further streamlining and integration of the devices by continuously harvesting biomechanical energy. However, the current energy conversion efficiency only supports brief operation of pacemakers, which is insufficient for sustained clinical application. It may be a candidate to prepare 3D porous scaffolds/multilayer structures with multiple microscale interfaces to increase power density. More innovations in flexible microelectronics, soft bioelectronics, micro‐nano fabrication, and multimodal sensing strategies are also needed to develop high‐energy‐density, multifunctional self‐powered cardiovascular devices.^[^ ^194^ ^]^

### 5.4 Artificial Intelligence (AI) and Machine Learning

Moreover, the integration of AI and machine learning can further optimize the performance of electroactive systems. By developing algorithms capable of capturing multimodal signals, including biomarkers, mechanical signals, and electrical signals, these technologies are expected to enhance the SNR ratio of data processing, achieving higher precision and efficacy.^[^ ^195^ ^]^ Coupled with large population data analysis, machine learning algorithms can assist in diagnostics and improve the efficiency of treatment plan formulation, significantly enhancing disease prediction capabilities.^[^ ^60^, ^196^ ^]^ Additionally, many studies concentrate on assessing drug effects by recording the electromechanical activity of cardiomyocytes in vitro.^[^ ^197^ ^]^ Such platforms facilitate the evaluation of cardiomyocyte electrophysiological responses to different drugs, and the assistance of AI allows for better development and screening of novel AAD. By analyzing large amounts of data from experiments and theoretical models, enables researchers to optimize the structure and properties of electroactive materials more efficiently and accurately,^[^ ^198^ ^]^ thus accelerating the development of materials and providing better options for personalized medical protocols.

### 5.5 Clinical Translation and Commercialization

The development of electroactive systems necessitates not only the innovative thinking of materials researchers but also a grounded understanding of clinical needs to facilitate bench‐to‐bedside translation. From a clinical translation perspective, electroactive materials exhibit significant commercialization potential by driving mass production and reducing manufacturing costs. However, regulatory challenges and patient acceptance of new technologies require careful consideration. Therefore, on the one hand, strict data encryption and access control measures are needed to ensure the privacy and security of patient health data.^[^ ^199^ ^]^ On the other hand, establishing comprehensive legal regulations is equally crucial, especially clinical guidelines. Future research should not only focus on optimizing technologies but also on how to transform these emerging innovations into safe, cost‐effective, and sustainable clinical products.^[^ ^200^ ^]^

**Figure 10:**
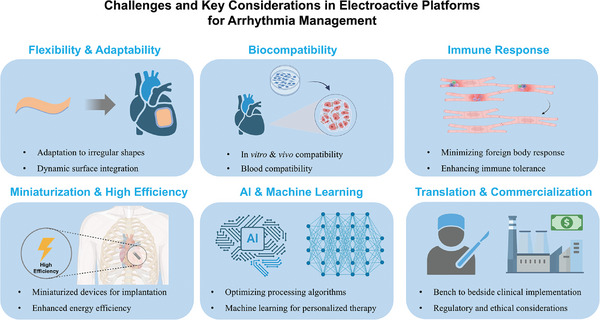
Key challenges and considerations for the development and clinical application of electroactive platforms in arrhythmia management.

We summarize the key considerations discussed above, as illustrated in **Figure** **10**. By continuously optimizing material properties, enhancing interdisciplinary collaboration, and advancing clinical translation, we are optimistic that technological advancements in this field will provide more precise, efficient, and personalized treatment options for patients with cardiac arrhythmias in the future.

## Conflict of Interest

The authors declare no conflict of interest.
